# Release from persistent T cell receptor engagement and blockade of aryl hydrocarbon receptor activity enhance IL-6-dependent mouse follicular helper T-like cell differentiation in vitro

**DOI:** 10.1371/journal.pone.0287746

**Published:** 2023-06-23

**Authors:** Rei Sakamoto, Ayumi Takada, Shinnosuke Yamakado, Haruki Tsuge, Etsuro Ito, Makoto Iwata

**Affiliations:** 1 Department of Biology, Waseda University, TWIns, Shinjuku, Tokyo, Japan; 2 Research Organization for Nano and Life Innovation, Waseda University, TWIns, Shinjuku, Tokyo, Japan; Universite Paris-Saclay, FRANCE

## Abstract

Follicular helper T (Tfh) cells are crucial for humoral immunity. Dysregulation of Tfh cell differentiation can cause infectious, allergic, and autoimmune diseases. To elucidate the molecular mechanisms underlying Tfh cell differentiation, we attempted to establish an in vitro mouse model of Tfh cell differentiation in the absence of other cell types. Various cytokines and cell surface molecules are suggested to contribute to the differentiation. We found that stimulating naïve CD4^+^ T cells with immobilized antibodies to CD3, ICOS, and LFA-1 in the presence of soluble anti-CD28 antibody, IL-6, and antibodies that block IL-2 signaling for 3 days induced the expression of *Bcl6* and *Ror*c*(γt)*, master regulator genes of Tfh and Th17 cells, respectively. TGF-β significantly enhanced cell proliferation and *Bcl6* and *Rorc(γt)* expression. An additional 2 days of culture without immobilized antibodies selectively downregulated *Rorc(γt)* expression. These cells produced IL-21 and promoted B cells to produce IgG antibodies. Adding the aryl hydrocarbon receptor (AhR) antagonist CH-223191 to the T cell culture further downregulated *Ror*c*(γt)* expression without significantly affecting *Bcl6* expression, and upregulated expression of a key Tfh marker, CXCR5. Although their CXCR5 expression levels were still not high, the CH-223191-treated cells showed chemotactic activity towards the CXCR5 ligand CXCL13. On the other hand, AhR agonists upregulated *Rorc(γt)* expression and downregulated CXCR5 expression. These findings suggest that AhR activity and the duration of T cell receptor stimulation contribute to regulating the balance between Tfh and Th17 cell differentiation. Although this in vitro system needs to be further improved, it may be useful for elucidating the mechanisms of Tfh cell differentiation as well as for screening physiological or pharmacological factors that affect Tfh cell differentiation including CXCR5 expression.

## Introduction

T cell-dependent antibody (Ab) responses have important roles in acquired immunity. Follicular helper T (Tfh) cells play a major role in helping B cells to differentiate into plasma cells in the germinal centers (GCs) of lymphoid organs [1, 2]. Tfh cells express the chemokine receptor CXCR5 that contributes to their migration into GCs [3]. The normal formation of GCs requires Tfh cells, and the development and function of Tfh cells are controlled by the transcription factor BCL6 [4–6]. Dysregulation of Tfh cell differentiation leads to various disorders including infectious diseases, autoimmune disorders, allergies, and possibly oncogenesis [1]. To clarify the precise molecular mechanisms underlying Tfh cell differentiation, not only in vivo studies but also in vitro studies with proper model systems are needed. Although in vitro models of human Tfh cell differentiation are established [7, 8], in vitro mouse models have much room for improvement or replacement [1, 9]. Thus, in the present study, we attempted to establish a new in vitro system for inducing the differentiation of mouse naïve CD4^+^ T cells almost simultaneously into Tfh cells in the absence of other cell types.

Previous in vitro studies on mouse Tfh cell differentiation have been typically based on stimulation of naïve CD4^+^ T cells with anti-CD3 and anti-CD28 Abs or with antigen-presenting cells [10–18]. Previous in vivo studies suggested that co-stimulatory signals not only via CD28 [19] but also via inducible T cell costimulator (ICOS, CD278) [16, 20, 21], lymphocyte function-associated antigen 1 (LFA-1, CD11a/CD18, αL/β2) [22], and OX40 (CD134, TNFRSF4) [23], enhance differentiation of mouse Tfh cells upon antigenic stimulation. Interestingly, it was shown that anti-ICOS stimulation can contribute to the early development of Tfh cells [16]. Pharmacological stimulation with phorbol myristate acetate and ionomycin also induces Tfh cell generation more efficiently than stimulation with anti-CD3 and anti-CD28 in the presence of IL-6 [24]. IL-6 is a potent inducer of murine Tfh cell differentiation [4, 11]. IL-6 and IL-21 redundantly induce mouse Tfh cell differentiation [10, 12, 25]. Both IL-6 and IL-21 promote STAT3 expression, and IL-6 induces IL-21 production in a STAT3-dependent manner [26–28]. In human, IL-12 is the most potent initiator of human Tfh cell program [29, 30]. IL-12 also induces Tfh cell-like phenotype in mouse cells in the early stage, but later induces Th1 phenotype [14]. However, when IL-2 is limited, IL-12 induces Tfh1-like cells that exhibit Tfh-like properties and can produce the Th1 cytokine IFN-γ in addition to IL-21 [17]. IL-2 inhibits Tfh cell differentiation by decreasing the expression of BCL6 and increasing the expression of the BCL6 antagonist BLIMP-1 (encoded by *Prdm1*) via STAT5 signaling [15, 31, 32]. IL-2 also represses IL-6 receptor (R) α and gp130 expression, and thus IL-6 responsiveness [33], although Tfh cells can produce IL-2 by themselves [34]. On the other hand, IL-6 inhibits T cell receptor (TCR)-mediated upregulation of IL-2Rβ (CD122) expression by preventing the association of STAT5 with the *Il2rb* locus [35]. IL-2Rα (CD25) expression is suppressed by TGF-β [36]. TGF-β provides critical additional signals for promoting the initial differentiation programs in human Tfh cells, but its effect on mouse Tfh cell differentiation remains controversial [4, 7, 8, 36]. It is well known that combining TGF-β and IL-6 or TGF-β and IL-21 induces Th17 cell differentiation and expression of its master regulator *Rorc(γt)* in a STAT3-dependent manner [27, 37]. In the present study, we found that TGF-β significantly enhanced the expression of both *Bcl6* and *Rorc(γt)* in mouse naïve CD4^+^ T cells stimulated with IL-6 and Abs to CD3 and costimulatory molecules including ICOS in the presence of Abs blocking IL-2 signaling. Subsequent culture without the CD3-mediated stimulation, however, selectively led to downregulated *Rorc(γt)* expression.

Th17 polarization is enhanced by stimulation of the aryl hydrocarbon receptor (AhR), a ligand-dependent transcription factor that responds to various polycyclic aromatic hydrocarbons [38–40]. AhR agonists can be generated from tryptophan present in the culture medium [41] and enhance Th17 cell differentiation [38–40, 42]. Furthermore, AhR is functionally upregulated early in the course of T-cell activation [43]. Here we found that addition of an AhR antagonist into the culture further downregulated *Rorc(γt)* expression and unexpectedly increased CXCR5 expression. The in vitro system that we established in the present study may provide a useful basis for analyzing further detailed mechanisms of Tfh cell differentiation and screening the effects of molecules and conditions on the Tfh cell differentiation.

## Materials and methods

### Mice and reagents for culture

Mice were obtained from Japan SLC and maintained in specific pathogen-free conditions in our animal facility. All animal experiments were performed according to the protocols approved by the Institutional Animal Care and Use Committee at Waseda University (#2017-A007a, 2018-A090, 2019-A041, 2020-A057, 2021-A003, A22-007, and A23-007). We used 7- to 12-wk-old male or female C57BL/6 mice. Mouse rIL-1β, rIL-2, rIL-4, rIL-6, rIL-21, rIL-23, rTGF-β1, rCXCL13, and anti-ICOS (clone C398.4A) monoclonal Abs (mAbs) were obtained from BioLegend. Mouse rIL-12 and Cellstain CFSE (5- or 6-(*N*-Succinimidyloxycarbonyl)fluorescein 3’,6’-diacetate) were from Fujifilm Wako Pure Chemistry. mAbs to CD28 (clone 37.51), IL-2 (clone JES6-1A12), IL-4 (clone 11B11), IL-2Rα (clone PC-61.5.3), IL-2Rβ (clone TM-β1), and IFN-γ (clone XMG1.2) were from Bio X Cell. CH-223191, 6-formylindolo[3,2-*b*]carbazole (FICZ) and 2-(1′H-indole-3′-carbonyl)-thiazole-4-carboxylic acid methyl ester (ITE) were from Cayman Chemical.

### Cell purification and culture

Naïve CD4^+^ T cells were purified from spleens by negative selection using EasySep Mouse CD4^+^ T Cell Enrichment kits (Stemcell Technologies) supplemented with biotinylated mAbs to mouse CD44 (clone IM7) and CD25 (clone PC61) (BioLegend), and subsequently by positive selection with CD62L Microbeads (Miltenyi Biotec). The purity was more than 96%. The purified cells were suspended (1.5–2 × 10^5^ cells/ml) in DMEM supplemented with 100 μM non-essential amino acids, 2 mM L-glutamine, 1 mM sodium pyruvate, 50 μM 2-mercaptoethanol, 20 mM HEPES (pH 7.2), 100 U/ml penicillin, 100 μg/ml streptomycin, and 10% FCS (cDMEM), and cultured in 96- or 48-well flat-bottom plates (MS-8096R or MS-8048R, Sumitomo Bakelite) coated with anti-CD3 mAb (clone 145-2C11) and other indicated mAbs in the presence of soluble anti-CD28 mAb under 10% CO_2_. Typically, T cells were stimulated with immobilized mAbs to CD3 (3 μg/ml), ICOS (1.5 μg/ml), and LFA-1 (CD11a) (clone M17/4) (1.5 μg/ml) in the presence of soluble anti-CD28 mAb (1 μg/ml) and IL-6 (20 ng/ml) for 3 days. In some experiments, the cells were further cultured in either the same well receiving an equal volume of fresh medium containing the indicated cytokines and Abs or a new culture well coated with or without Abs to CD3, ICOS, and LFA-1 receiving an equal or the indicated volume of medium or replacing the culture supernatant with fresh medium supplemented with the indicated cytokines and blocking Abs. However, when the supernatant of the first culture was carried over to the second culture, further addition of blocking Abs had little effect on Tfh-like cell differentiation. In some experiments, the AhR antagonist CH-223191 or the AhR agonists FICZ or ITE were added to the culture. These reagents were dissolved in DMSO, but final DMSO concentrations were less than 0.1%, and did not significantly affect the results.

To induce Th1 cells, naïve CD4^+^ T cells (3 × 10^4^ cells) were cultured in 0.2 ml of cDMEM containing IL-12 (10 ng/ml) and anti-IL-4 (10 μg/ml) in 96-well plates (Sumilon) coated with 3 μg/ml of anti-CD3 and 3 μg/ml of anti-CD28. After 3 days, the cells were resuspended in 0.5 ml/well fresh cDMEM containing IL-12 (10 ng/ml) and IL-2 (50 U/ml), transferred to 48-well culture plates (Falcon), and cultured for 2 days. To induce Th2 cells, naïve CD4^+^ T cells (3 × 10^4^ cells) were cultured in 0.2 ml of cDMEM containing IL-4 (40 U/ml) and anti-IFN-γ (10 μg/ml) in 96-well plates coated with 3 μg/ml of anti-CD3 and 3 μg/ml of anti-CD28. After 3 days, the cells were resuspended in 0.5 ml/well fresh medium containing IL-4 (40 U/ml) and IL-2 (50 U/ml), transferred to 48-well plates, and cultured for 2 days. To induce Th17 cells, naïve CD4^+^ T cells (3 × 10^4^ cells) were cultured in 0.2 ml of cDMEM containing TGF-β1 (4 ng/ml), IL-1β (10 ng/ml), IL-6 (20 ng/ml), IL-21 (10 ng/ml), IL-23 (10 ng/ml), TNF-α (20 ng/ml), anti-IFN-γ (10 μg/ml), anti-IL-2 (10 μg/ml), anti-CD25 (10 μg/ml), anti-CD122 (10 μg/ml), and anti-IL-4 (10 μg/ml) in 96-well plates coated with 3 μg/ml of anti-CD3 and 3 μg/ml of anti-CD28. After 3 days, the cultures were transferred to anti-CD3/anti-CD28-coated 48-well culture plates (without removing the culture supernatant) and received 0.3 ml/well fresh medium containing TGF-β1 (4 ng/ml), IL-1β (10 ng/ml), IL-6 (20 ng/ml), IL-21 (10 ng/ml), IL-23 (10 ng/ml), and TNF-α (20 ng/ml), and further cultured for 2 days. To induce Th0 cells, naïve CD4^+^ T cells (3 × 10^4^ cells) were cultured in 0.2 ml of the medium containing anti-IFN-γ (10 μg/ml) and anti-IL-4 (10 μg/ml) in 96-well plates (Sumilon) coated with 3 μg/ml of anti-CD3 and 3 μg/ml of anti-CD28. After 3 days, the cells were resuspended in 0.5 ml/well fresh medium containing anti-IFN-γ (10 μg/ml) and anti-IL-4 (10 μg/ml) and IL-2 (50 U/ml), transferred to 48-well culture plates (Falcon), and cultured for 2 days. In some experiments, the Th cells (1 × 10^5^ cells) were washed and cultured in 0.2 ml/well of cDMEM in 96-well plates coated with 3 μg/ml of anti-CD3 and 3 μg/ml of anti-CD28 for 2 days, and the culture supernatants were obtained for assessing the cytokine production by ELISA. For intracellular staining of cytokines, the cells were cultured as for ELISA, and monensin (2 μM, Cayman) was added for the last 2 hours of the culture.

In some experiments, RPMI 1640 medium was used in place of the DMEM, and the cells were cultured under 5% CO_2_.

### Real-time PCR

Total RNA was isolated from cells using a ReliaPrep RNA Cell Miniprep System (Promega) or RNeasy Mini Kit (Qiagen), and cDNA was generated using ReverTra Ace qPCR RT Master Mix with gDNA Remover (Toyobo). cDNA was used as a template for real-time PCR with BrightGreen qPCR MasterMix-ROX (Applied Biosystems) and gene-specific primers. PCR and analysis were performed on a StepOnePlus Real-Time PCR system (Applied Biosystems). The relative expression of each gene was quantified with the 2^−ΔCt^ value multiplied by 1000, where ΔCt was the difference between the mean Ct value of triplicates or quadruplicates of the sample and that of the endogenous *Rplp0* control [44]. Sequences of the primers for *Rplp0*, *Bcl6*, *Rorc(γt)*, *Prdm1*, *Tbx21*, *Gata3*, *Ahr*, and *Cyp1a1* are listed in S1 Table in S1 File.

### Flow cytometric analysis

The cells were stained with mAb to CXCR5 (CD185) (clone L138D7) APC or Alexa Fluor 647, or together with mAbs to PD-1 (CD279) (clone 29F.1A12) FITC and ICOS (CD278) (clone C398.4A) PE or with mAbs to CCR7 (CD197) (clone 4B12) Alexa Fluor 488 and CXCR4 (CD184) (clone QA16A08) PE or with mAbs to CD138 (Syndecan-1) (clone 281–2) PE, CD45R (B220) (clone RA3-6B2) APC, and CD4 (clone RM4-5) FITC in the presence of anti-CD16/CD32 (clone 2.4G2) mAb (all from BioLegend). Stained cells were analyzed with a BD Accuri^TM^ C6 Plus Flow Cytometer (BD). Live cells were gated based on the forward scatter (FSC) and side scatter (SSC) and the exclusion of dead cells using 7-amino-actinomycin D (7-AAD). Doublets were excluded by using the FSC-A/FSC-H gating. Typical gating strategies for flow cytometric analysis of cultured cells are shown in S1 Fig in S1 File. In some experiments, antigen expression levels were expressed as Δ mean fluorescence intensity (ΔMFI), which was calculated as: (MFI of the cells stained with a fluorochrome-conjugated Ab)–(MFI of the isotype control Ab staining).

Intracellular staining was performed with anti-BCL6 (clone K112-91) PE and anti-RORγt (Q31-378) Alexa Fluor 647 (both from BD Biosciences) or with anti-IL-21 (clone FFA21) PE (eBioscience) and anti-IL-17A (clone TC11-18H10.1) APC (BioLegend) using Foxp3/Transcription Factor Staining Buffer Sets (eBioscience) according to the manufacturer’s instructions.

### Cytokine ELISA

Cytokine levels in the culture supernatants were analyzed with ELISA kits for IL-21 (R&D Systems).

### CFSE labeling and cell proliferation analysis

Naïve CD4^+^ T cells were labeled with CFSE (5 μM) for 5 minutes at room temperature as previously described [45]. The cells were washed three times and cultured in the Tfh-like cell-inducing conditions in the presence or absence of TGF-β1 (0.1 or 1 ng/ml). After the culture, the cells were analyzed for their CFSE fluorescence intensity.

### T and B cell co-culture

Indicated helper T cells were incubated with mitomycin C (30 μg/ml; Wako) at 37°C for 30 minutes. B cells were purified from the spleen of age- and sex-matched C57BL/6 mice by negative selection using EasySep Mouse B Cell Isolation kits (Stemcell Technologies) according to the manufacturer’s instructions. Mitomycin C-treated T cells (1.5 × 10^5^ cells) were extensively washed and co-cultured with purified B cells (1.5 × 10^5^ cells) in the presence of soluble anti-CD3 mAb (0.1 μg/ml) (BioLegend) in 0.2 ml of the medium (RPMI 1640) in 96-well round bottom plates (Falcon) for 7 days. The culture supernatant was analyzed for total IgG Ab production by using IgG (Total) Mouse Uncoated ELISA kits (Invitrogen) according to the manufacturer’s instructions. OD450 values (ΔOD450) were calculated by subtracting OD450 readings taken from supernatants from B cells cultured alone from the OD450 values of co-cultured samples as previously reported [17]. In some experiments, the cultured cells were stained with anti-CD138 PE, anti-CD45R/B220 APC, and anti-CD4 FITC for excluding CD4^+^ T cells and detecting plasma cells.

### Chemotaxis assay

To examine if the chemokine receptor CXCR5 expressed on T cells was functionally active, transwell chemotaxis assays were performed as previously described [46] with a slight modification. Briefly, 0.6 ml of 10% FCS (RPMI 1640) with or without 1 μg/ml CXCL13 was added into the lower well of the Transwell plates (Corning 3421), and 5 × 10^5^ cells suspended in 0.1 ml of 10% FCS (RPMI 1640) were added into the upper well and incubated for 3 hours at 37°C. The numbers of migrated cells into the lower wells were counted, and their percentages relative to the input cell number were calculated.

### Immunization of mice for Tfh cell induction

For analyzing Tfh cells in vivo, each C57BL/6 mouse was immunized by intraperitoneal (i.p.) injection with ovalbumin (OVA; 100 μg) and lipopolysaccharide (LPS; 10 μg) (both from Sigma-Aldrich) in aluminium hydroxide gel (Alum; 2 mg) (Wako) as described previously [47]. After 8 days, CD4^+^ T cells were purified from spleens and mesenteric lymph nodes by negative selection using EasySep Mouse CD4^+^ T Cell Enrichment kits. Their expression of CXCR5, PD-1, BCL6 and RORγt protein was determined by flow cytometry.

### Statistical analysis

One-way ANOVA with Tukey-Kramer multiple comparison test or the Student unpaired two-tailed *t* test was used to analyze differences between conditions. Values of *p* < 0.05 were considered significant. We performed statistical analyses with biological replicates, and “n” represents the number of culture wells or animals.

## Results

### Blocking of IL-2 signaling enhances the expression of *Bcl6* and *Rorc(γt)* in vitro

**Fig 1:**
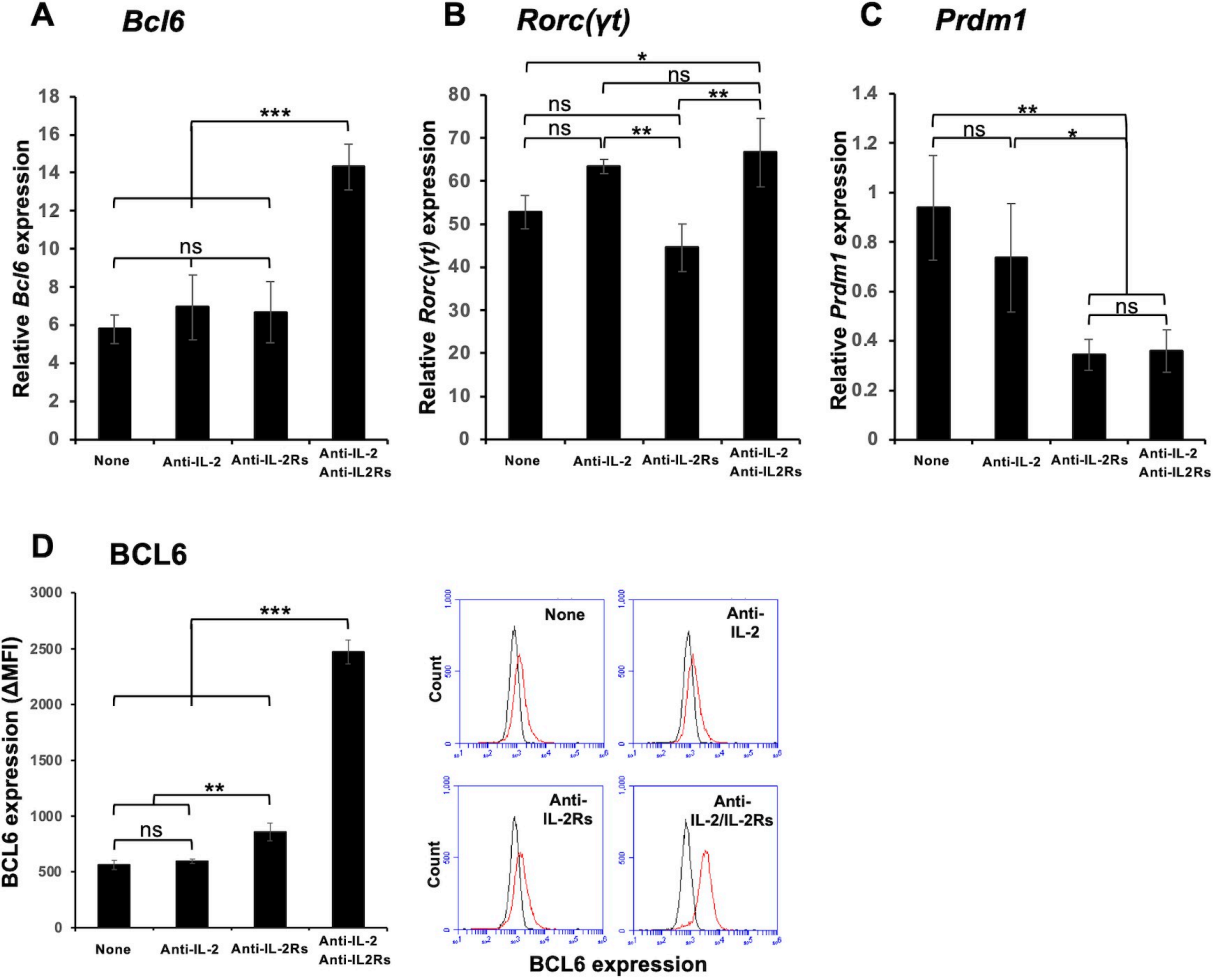
Addition of blocking Abs to IL-2Rα and IL-2Rβ enhances *Bcl6* expression. Naïve CD4^+^ T cells were stimulated with immobilized mAbs to CD3, ICOS, and LFA-1 (3, 1.5, and 1.5 μg/ml for coating, respectively) in the presence of soluble anti-CD28 mAb (1 μg/ml), IL-6 (20 ng/ml), and neutralizing mAbs to IL-2 (10 μg/ml) and IL-4 (10 μg/ml) with or without blocking mAbs to IL-2Rα (CD25; 10 μg/ml) and IL-2Rβ (CD122; 10 μg/ml) for 3 days in DMEM. Relative mRNA expression of (**A**) *Bcl6*, (**B**) *Rorc(γt)*, and (**C**) *Prdm1* was analyzed by real-time PCR. (**D**) Intracellular expression of BCL6 protein was analyzed by flow cytometry after fixation and permeabilization. Representative flow cytometry histograms are also shown. Data are presented as mean ± SD of quadruplicate or triplicate samples. Results shown are representative of three independent experiments. *p < 0.05, **p < 0.01, ***p < 0.001. ns, not significant.

To identify the optimal conditions for inducing Tfh cell differentiation in the absence of other cell types in vitro, we stimulated isolated mouse naïve CD4^+^ T cells with immobilized antibodies to CD3 and various co-stimulatory molecules in the presence of soluble anti-CD28 Ab, IL-6, and neutralizing Abs to some cytokines for 3 days. We found that the combination of immobilized Abs to CD3, ICOS, and LFA-1 and a neutralizing Ab to IL-4 consistently induced the expression of both *Bcl6* and *Rorc(γt)* (Fig 1A and 1B). We also added a neutralizing Ab to IL-2 and blocking Abs to IL-2Rα (CD25) and IL-2Rβ (CD122), as IL-2 is a critical regulator of Tfh cell differentiation [31]. The combination of Abs to IL-2 and IL-2 receptors (IL-2Rs) significantly enhanced the expression of *Bcl6* mRNA and BCL6 protein (Fig 1A and 1D), and moderately enhanced *Rorc(γt)* expression (Fig 1B). Expression of *Prdm1*, which encodes the BCL6 antagonist BLIMP-1 was downregulated by anti-IL-2Rs with or without anti-IL-2 (Fig 1C). For comparison, we activated naïve CD4^+^ T cells under the Th1, Th2, or Th17 cell-inducing condition for 3 days. These cells expressed significantly lower levels of *Bcl6* expression (S2 Fig in S1 File). These results suggest that strict restriction of IL-2 signaling is indeed crucial for enhancing *Bcl6* expression for Tfh-like cell differentiation, but that additional factors or conditions are required to suppress *Rorc(γt)* expression.

### Release from persistent TCR engagement selectively downregulates *Rorc(γt)* expression

**Fig 2:**
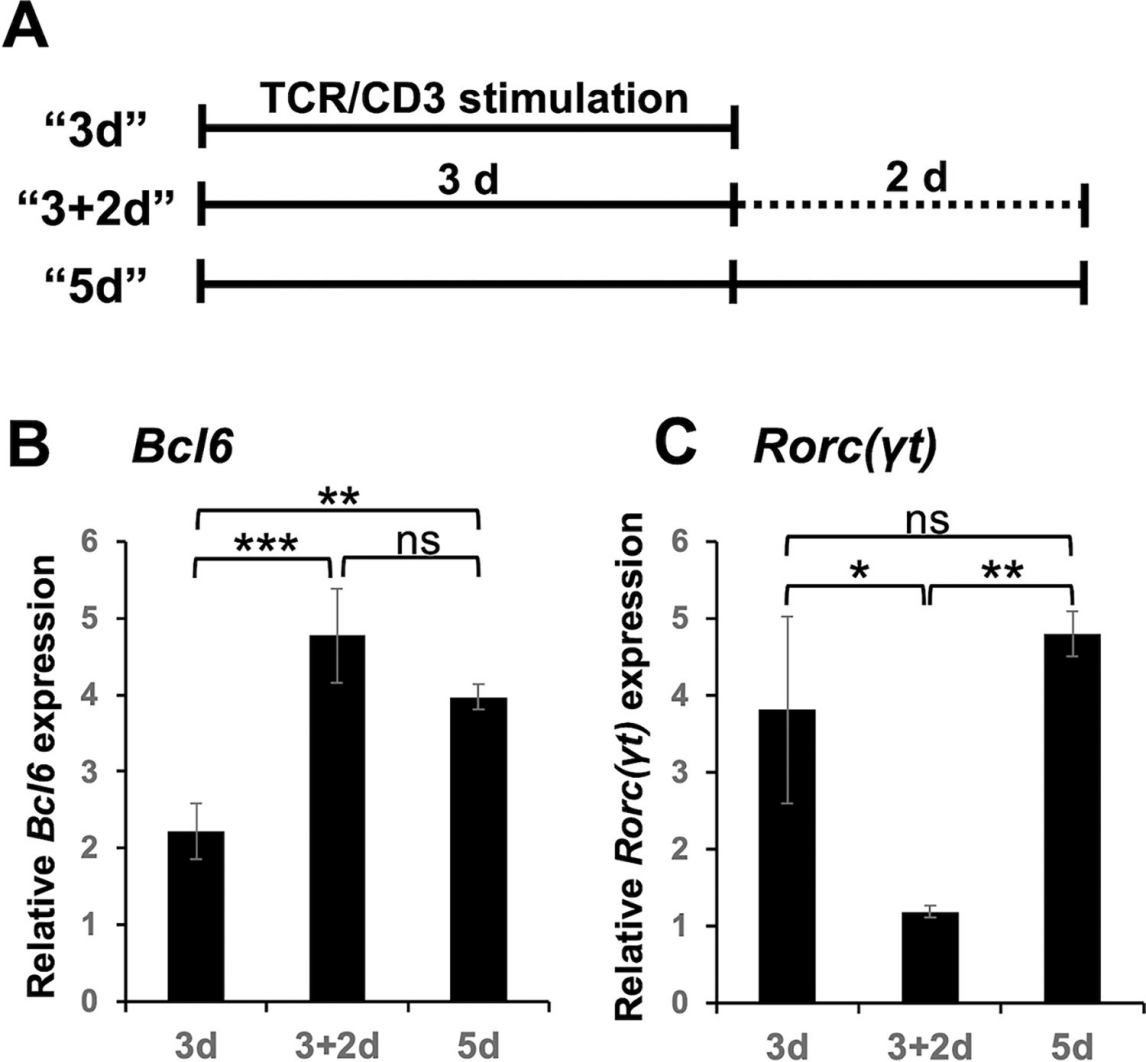
The release from persistent TCR stimulation reciprocally regulates *Bcl6* expression and *Rorc(γt)* expression. Naïve CD4^+^ T cells were stimulated as described in the legend of Fig 1 in the presence of blocking mAbs to IL-2Rs for 3 days (“3d”). Aliquots of cells were transferred to a new well without Abs to CD3, ICOS, and LFA-1 receiving an equal volume of fresh DMEM supplemented with IL-6 (20 ng/ml), anti-IL-2 (10 μg/ml), and anti-IL-2Rs (10 μg/ml each) and were further cultured for 2 days (“3+2d”). Other aliquots of cells received an equal volume of fresh DMEM supplemented as above in the same well, and further cultured for 2 days (“5d”) as schematically shown in (**A**). After the culture, relative mRNA expression of (**B**) *Bcl6* and (**C**) *Rorc(γt)* were analyzed by real-time PCR. Data are presented as mean ± SD of triplicate samples. Results shown are representative of three independent experiments. *p < 0.05, **p < 0.01, ***p < 0.001. ns, not significant.

Tfh cell differentiation in vivo appears to be initiated by interactions with antigen-presenting DCs in lymphoid organs and to proceed after detaching from DCs and entering into B cell-rich follicles [1]. Accordingly, Powell et al. induced “Tfh0-like” cells after 3 days of stimulation of naïve CD4^+^ T cells with Abs to CD3 and CD28 in the presence of IL-6 and Abs to IFN-γ and IL-4, followed by 2 days of incubation without the stimulation in the presence of low IL-2 [17]. We hypothesized that release from the first TCR engagement might be required to promote the differentiation. Thus, after 3 days of culture in Ab-coated culture plates (1^st^ “3d” culture), aliquots of cells were transferred and cultured for 2 more days (2^nd^ culture) in new plates without immobilized Abs (“3+2d” culture), and other aliquots of cells were continuously cultured in the original wells for 2 more days (“5d” culture) (Fig 2A). Compared with the “3d” culture, the 2-step “3+2d” culture significantly upregulated *Bcl6* expression (Fig 2B) and downregulated *Rorc(γt)* expression (Fig 2C). However, the “5d” culture did not downregulate *Rorc(γt)* expression (Fig 2C) and upregulated *Bcl6* expression (Fig 2B). The *Bcl6* expression after the “5d” culture was tended to be lower than that after the “3+2d” culture, although not significantly. The results suggest that the release from the persistent TCR stimulation shifts the balance of differentiation away from the Th17 cell fate toward Tfh-like cell generation.

### TGF-β enhances the expression of *Bcl6* and *Rorc(γt)*

**Fig 3:**
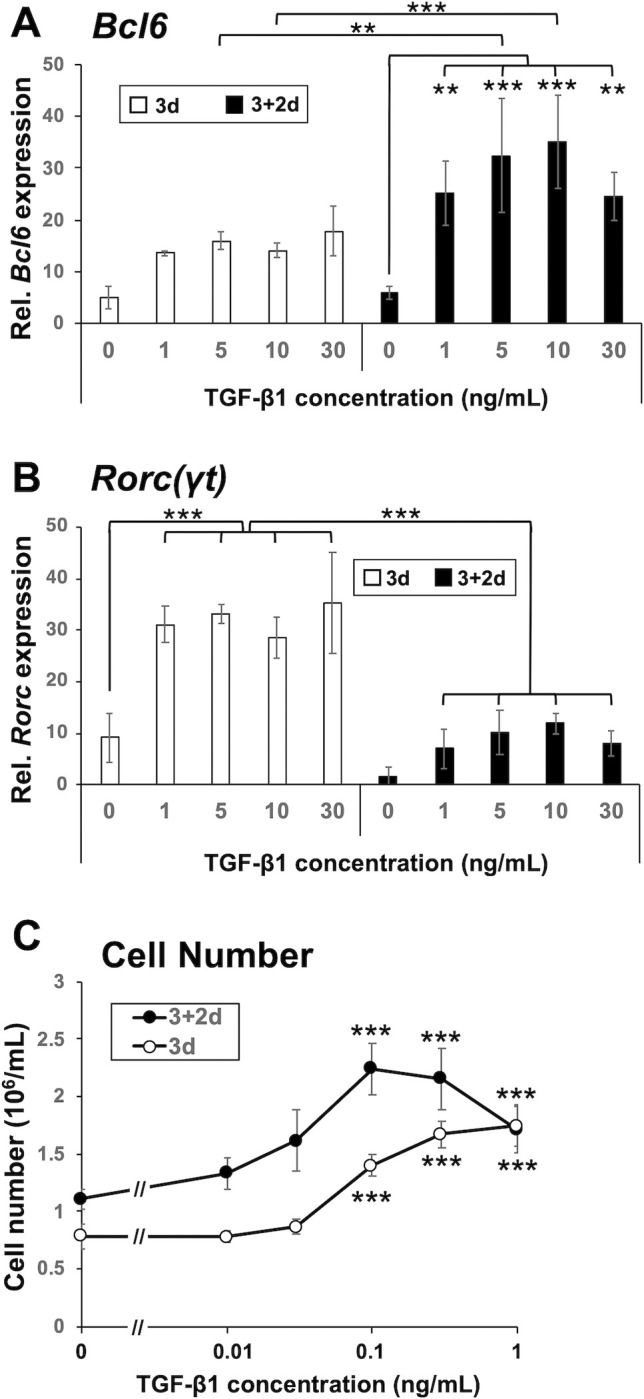
TGF-β enhances *Bcl6* expression and cell proliferation. Naïve CD4^+^ T cells were stimulated for 3 days as described in the legend of Fig 2 but in the presence of graded concentrations of TGF-β1 in DMEM (“3d”). Aliquots of cells were transferred to a new well without immobilized Abs, receiving an equal volume of fresh DMEM supplemented with IL-6, graded concentrations of TGF-β1, and blocking Abs to IL-2 signaling, and were further cultured for 2 days (“3+2d”). After the “3d” culture (*open bar*) and the “3+2d” culture (*closed bar*), relative mRNA expression of (**A**) *Bcl6* and (**B**) *Rorc(γt)* was analyzed by real-time PCR. (**C**) Recovered cell number from the “3d” culture and the “3+2d” culture is shown. Stars indicate that the cell number in the culture with TGF-β1 was significantly different from that in the culture without adding TGF-β1. Data are presented as mean ± SD of quadruplicate samples. Results shown are representative of three independent experiments. **p < 0.01, ***p < 0.001. ns, not significant.

TGF-β provides critical additional signals that promote the initial differentiation programs in human Tfh cells, but its effect on mouse Tfh cell differentiation remains controversial [4, 7, 8, 36]. We thus examined the effect of TGF-β1 on the T cell differentiation in the present in vitro system. TGF-β1 significantly enhanced the expression of *Bcl6* (Fig 3). The combination of IL-6 and TGF-β1 induces Th17 cell differentiation [48]. Accordingly, TGF-β1 also enhanced *Rorc(γt)* expression, but the expression was significantly lower after the “3+2d” culture than after the “3d” culture. In contrast, the *Bcl6* expression was significantly higher after the “3+2d” culture than after the “3d” culture. TGF-β1 also increased cell number even at 0.1 ng/ml after both the “3d” culture and the “3+2d” culture (Fig 3C). We thus performed CFSE-based proliferation assays. As shown in S3 Fig in S1 File, we confirmed an enhanced proliferation in TGF-β1-treated cells after the “3d” culture. However, after the “3+2d” culture, CFSE signals were not significantly affected with TGF-β1. The FSC/SSC plots suggested that TGF-β1 suppressed the cell death (S3A Fig in S1 File). TGF-β1 significantly suppressed ICOS expression after the “3d” culture as reported [7], but only moderately suppressed the expression after the “3+2d” culture (S3B Fig in S1 File). The results suggest that TGF-β1 potentiates the differentiation, proliferation, and survival of Tfh-like cells.

### BCL6 protein expression is not parallel to its mRNA expression

**Fig 4:**
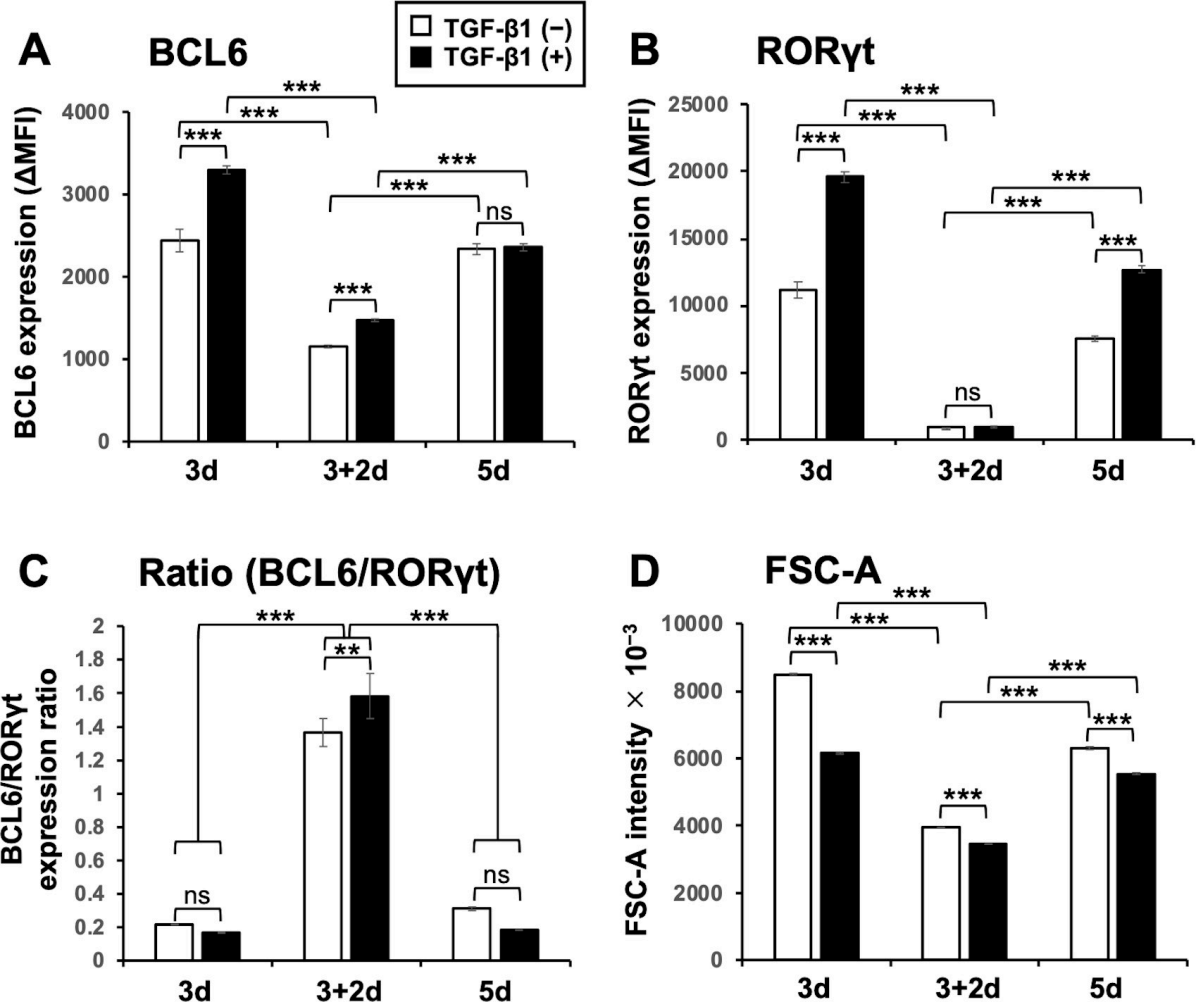
The release from persistent TCR stimulation reduces the expression of BCL6 protein and the cell size, and more effectively reduces the expression of RORγt protein. Naïve CD4^+^ T cells were stimulated as described in the legend of Fig 2 in the presence or absence of added TGF-β1 (1 ng/mL). After the culture, aliquots of cells were fixed and permeabilized, and their intracellular expression of (**A**) BCL6 protein and (**B**) RORγt protein was analyzed by flow cytometry, and their ΔMFI ratios are shown (**C**). (**D**) Forward scatter (FSC)-A intensities of the cells before fixation/permeabilization are shown. Data are presented as mean ± SD of triplicate or quadruplicate samples. Results shown are representative of three independent experiments. **p < 0.01, ***p < 0.001. ns, not significant.

We also analyzed the protein expression of BCL6 and RORγt after the “3d”, “3+2d”, and “5d” cultures in the presence or absence of added TGF-β1 (1 ng/mL). Surprisingly, the protein expression of BCL6 was not parallel to the mRNA expression (Fig 4A and S4 Fig in S1 File). The protein expression of BCL6 after the “3+2d” culture was apparently lower than that after the “3d” or “5d” culture. On the other hand, the protein expression of RORγt was nearly parallel to its mRNA expression (Fig 4B and S4 Fig in S1 File). Indeed, the ΔMFI ratio of BCL6 and RORγt expression after the “3+2d” culture was much higher than that after the “3d” or “5d” culture (Fig 4C). However, the effects of TGF-β1 on both BCL6 and RORγt protein expression were similar to those observed on their mRNA expression (Fig 4 and S4 Fig in S1 File). Interestingly, the FSC intensity after the “3+2d” culture was much lower than that after the “3d” or “5d” culture (Fig 4C and S4 Fig in S1 File). TGF-β1 further downregulated the FSC intensity. The results suggest that the cells after the “3+2d” culture were much smaller than those immediately after the persistent TCR stimulation. Therefore, after the release from the persistent TCR stimulation, the concentration of BCL6 protein in the cells might not be largely affected, whereas RORγt protein levels might be significantly downregulated. TGF-β1 appeared to enhance the concentration of BCL6 protein as well in these cells. Therefore, we took advantage of the “3+2d” culture condition for improving the in vitro model system in the following experiments. The discrepancy between mRNA expression and protein expression of BCL6 especially after the “3d” culture may be partly due to the repressor function of BCL6 that can repress even its own gene (9).

### Tfh-like cells are distinct from Th1 and Th2 cells, but share some features common to Th17 cells

The Tfh-like cells expressed *Bcl6* and *Rorc(γt)*, but not the master regulator genes of Th1 and Th2, *Tbx21* and *Gata3*, respectively (S5 Fig in S1 File). Th1 and Th2 cells did not significantly express *Bcl6* or *Rorc(γt)*. Th17 cells also exhibited *Bcl6* expression but at lower levels than Tfh-like cells and higher levels of *Rorc(γt)* expression. Tfh cells as well as Th2 and Th17 cells produce IL-21 [27, 28, 37, 49, 50]. The present Tfh-like cells produced IL-21, but at lower levels than Th17 cells induced under the present condition (S5F Fig in S1 File). However, intracellular staining for IL-21 indicated that Tfh-like cells and Th17 cells were producing similar levels of IL-21 after 2 days of CD3/CD28-mediated stimulation (S6 Fig in S1 File). As Tfh-like cells were rested for 2 days before restimulation, there might be a delay in the production of IL-21 compared to Th17 cells. On the other hand, IL-17A production by Tfh-like cells was much less than that by Th17 cells (S6 Fig in S1 File). These results collectively suggest that the generated Tfh-like cells are distinct from Th1, Th2, and Th17 cells, but still share some features common to Th17 cells.

### Replacing the DMEM with RPMI 1640 medium induces the downregulation of RORγt expression

**Fig 5:**
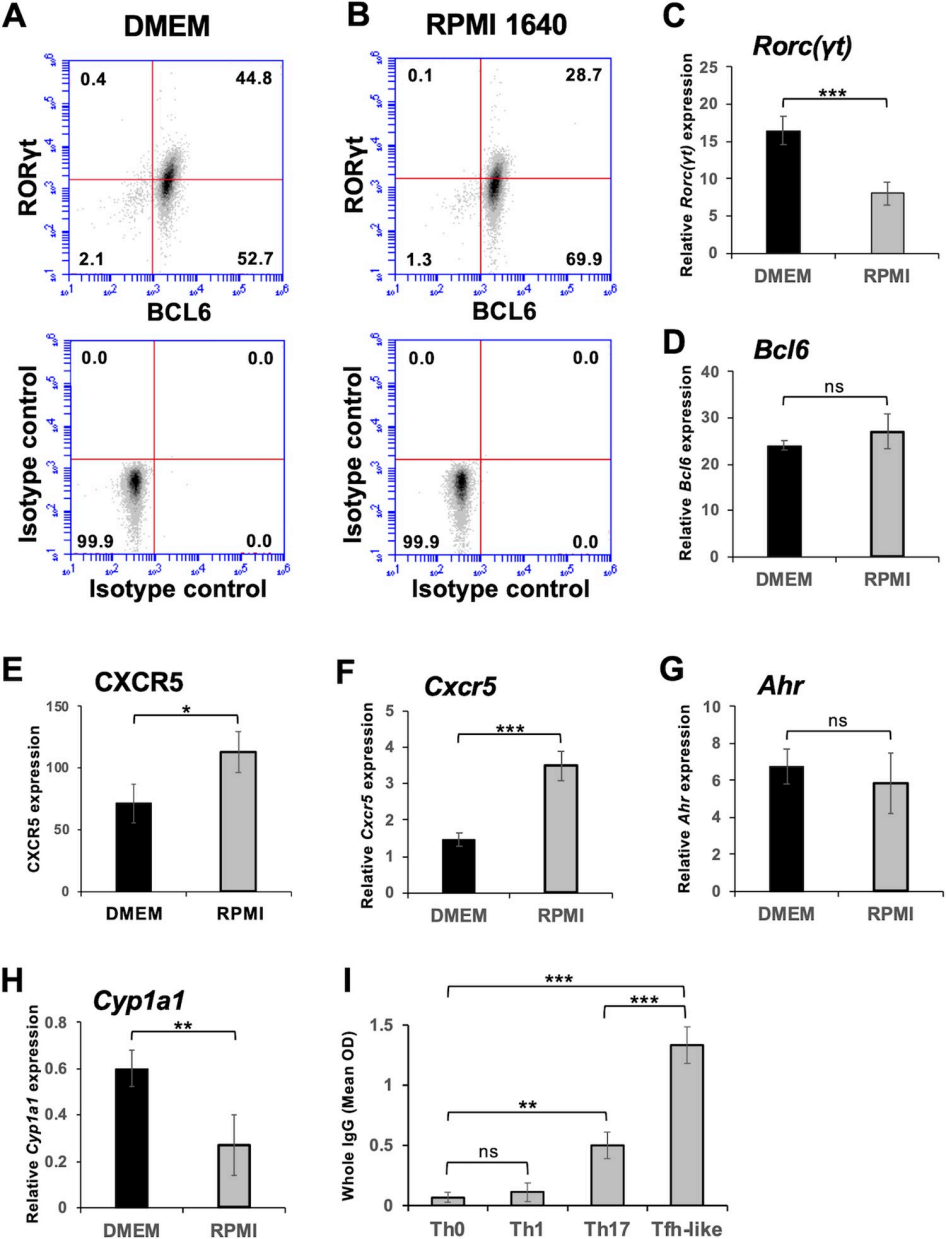
Reciprocal regulation of *Cxcr5* expression and *Rorc(γt)* expression by replacing DMEM with RPIMI 1640 medium, and Tfh-like cells generated in RPMI 1640 activate B cells to produce IgG. Tfh-like cells were induced from naïve CD4^+^ T cells by the “3+2d” culture with TGF-β1 (1 ng/ml) as described in the legend of Fig 3 but using either DMEM (*black closed bar*) or RPMI 1604 medium (*gray bar*). Th0, Th1 and Th17 cells were induced from naïve CD4^+^ T cells in the RPMI 1640 medium as described in Materials and Methods. (**A** and **B**) Representative flow cytometry density plots of intracellular expression of Bcl6 and RORγt in (A) DMEM culture and (B) RPMI 1640 culture. The quadrants are based on the isotype control Abs, and the numbers in each quadrant indicate the percentage of cells in the quadrant. Relative mRNA expression of (**C**) *Rorc(γt)*, (**D**) *Bcl6*, (**F**) *Cxcr5*, (**G**) *Ahr*, and (**H**) *Cyp1a1* was analyzed by real-time PCR. Cell surface expression of I CXCR5 was analyzed by flow cytometry. Data are presented as mean ± SD of quadruplicate samples. (**I**) The indicated helper T cells were treated with mitomycin C and cultured with B cells (1:1 T/B ratio) for 7 days. Production levels of whole IgG, assessed by ELISA, are shown. Data are presented as mean ± SD of triplicate samples. Results shown are representative of at least three independent experiments. *p < 0.05, **p < 0.01, ***p < 0.001. ns, not significant.

Most of the Tfh-like cells generated at this stage expressed BCL6 protein and their significant population also expressed RORγt protein (Fig 5A). We noticed that replacing the DMEM with RPMI 1640 medium induced the downregulation of RORγt protein and mRNA expression (Fig 5A–5C), but did not significantly affect BCL6 protein and mRNA expression (Fig 5A, 5B, and 5D). Unexpectedly, replacing the medium slightly but significantly upregulated CXCR5 surface protein and mRNA expression (Fig 5E, 5F, and S7A Fig in S1 File). Tfh-like cells generated in the RPMI 1640 medium produced significantly higher levels of IL-21 and lower levels of IL-17A than Th17 cells (S7B and S7C Fig in S1 File), and significantly enhanced IgG production by B cells in vitro (Fig 5I). The IgG production levels were higher than those induced by Th0, Th1, and Th17 cells in T-B cell co-cultures. Flow cytometric analysis of the cultured cells indicated that Tfh-like cells induced CD138^high^B220^low^ plasma cells more efficiently than Th17 cells (S8 Fig in S1 File), while Th0 and Th1 cells induced almost no plasma cells. These results suggest that Tfh-like cells generated in the RPMI 1640 were functional helper T cells.

RPMI 1640 medium contains lower concentrations of aromatic amino acids than DMEM (S2 Table in S1 File). The aromatic amino acids present in culture medium can be or turn to be AhR agonists and enhance Th17 cell differentiation [42]. Thus, replacing DMEM to RPMI 1640 medium might cause reduced AhR activity in the cells. Although expression of *Ahr* was not significantly affected by changing the medium (Fig 5G), expression of the AhR-regulated gene *Cyp1a1* was significantly downregulated (Fig 5H), suggesting that the AhR activity was reduced by the RPMI 1640.

### AhR activity regulates the differentiation balance between Tfh-like cells and Th17 cells

**Fig 6:**
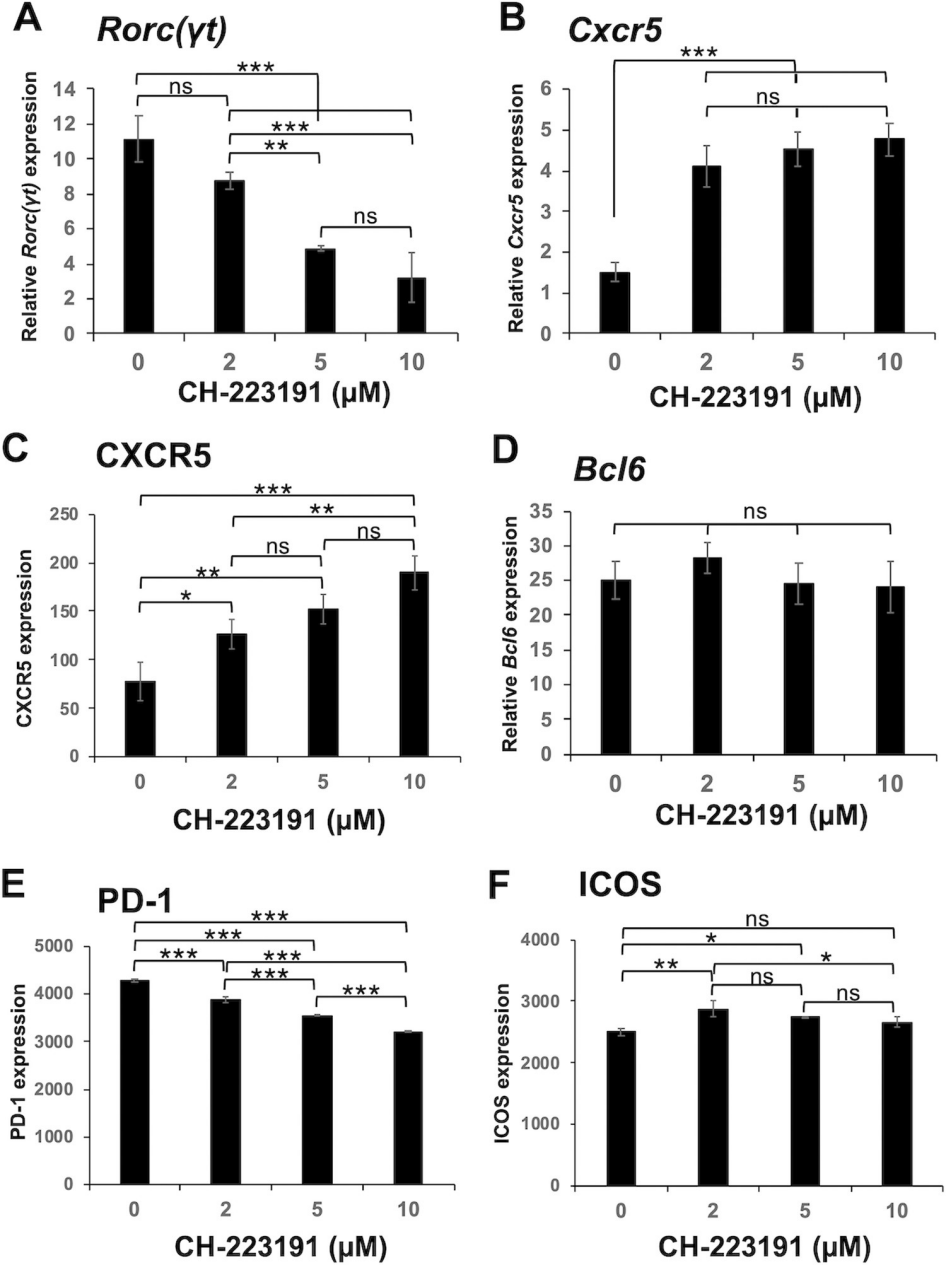
The AhR antagonist CH-223191 downregulates *Rorc(γt)* expression and upregulates *Cxcr5* mRNA expression and cell surface CXCR5 expression. Tfh-like cells were induced from naïve CD4^+^ T cells in DMEM as described in the legend of Fig 5 (“3+2d”) in the presence of graded concentrations of CH-223191. Relative mRNA expression of (**A**) *Rorc(γt)*, (**B**) *Cxcr5*, and (**D**) *Bcl6* was analyzed by real-time PCR. Cell surface expression of (**C**) CXCR5, (**E**) PD-1, and (**F**) ICOS was analyzed by flow cytometry. Data are presented as mean ± SD of triplicate samples. Results shown are representative of at least three independent experiments. *p < 0.05, **p < 0.01, ***p < 0.001. ns, not significant.

**Fig 7:**
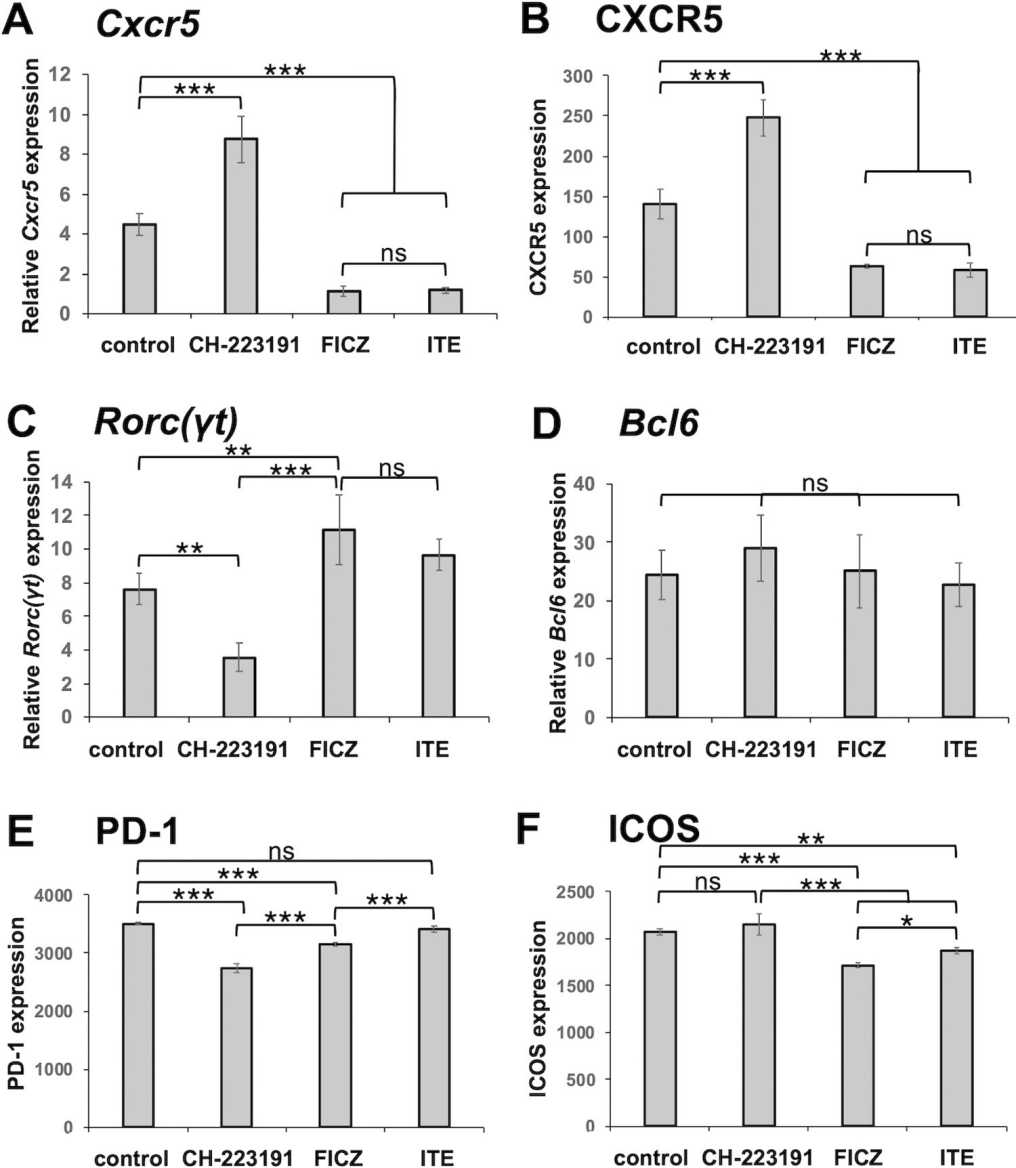
CH-223191 and the AhR agonists FICZ and ITE reciprocally regulate *Rorc(γt)* and *Cxcr5* expression. Naïve CD4^+^ T cells were cultured as described in the legend of Fig 5 (“3+2d”) in RPMI in the presence of CH-223191 (5 μM), FICZ (100 nM), ITE (1 μM), or vehicle control (**A**—**F**). Relative mRNA expression of (**A**) *Cxcr5*, (**C**) *Rorc(γt)*, and (**D**) *Bcl6* was analyzed by real-time PCR. Cell surface expression of (**B**) CXCR5, (**E**) PD-1, and (**F**) ICOS was analyzed by flow cytometry. Data are presented as mean ± SD of quadruplicate samples. Data are presented as mean ± SD of triplicate samples. Results shown are representative of at least three independent experiments. *p < 0.05, **p < 0.01, ***p < 0.001. ns, not significant.

Consistent with the above results, adding the AhR antagonist CH-223191 [42, 51] to the DMEM culture suppressed *Rorc(γt)* expression (Fig 6A) and enhanced *Cxcr5* and CXCR5 expression (Fig 6B, 6C, and S9 Fig in S1 File), but did not affect *Bcl6* expression (Fig 6D). Expression of PD-1 and ICOS was high and moderately affected by CH-223191 (Fig 6E, 6F, and S9 Fig in S1 File). In the RPMI culture, CH-223191 further enhanced *Cxcr5* and CXCR5 expression, while the AhR agonists FICZ [52] and ITE [53] significantly suppressed the expression (Fig 7A, 7B, and S10A Fig in S1 File). On the other hand, *Rorc(γt)* expression was significantly suppressed by CH-223191 but upregulated by FICZ (Fig 7C) as expected. ITE tended to upregulate the expression, but not significantly. *Bcl6* expression was also not significantly affected in RPMI 1640, and expression of PD-1 and ICOS was moderately or not significantly affected by the AhR modulators (Fig 7D–7F, and S10 Fig in S1 File).

**Fig 8:**
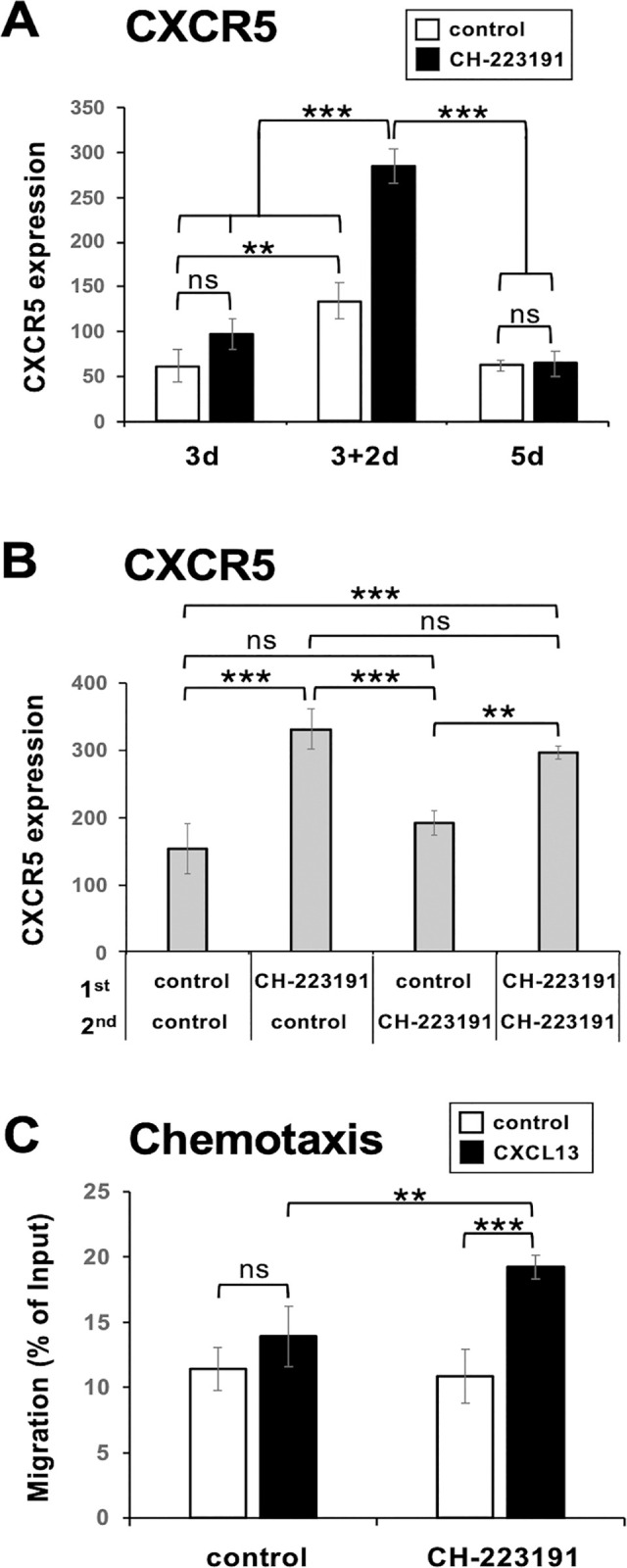
CH-223191 treatment during TCR engagement induces expression of functional CXCR5 after the release from TCR engagement. (**A**) Naïve CD4^+^ T cells were cultured as described in the legend of Fig 2 (“3d”, “3+2d”, and “5d”) but in RPMI medium containing TGF-β1 (1 ng/ml). CH-223191 (5 μM) or vehicle control was added in the first culture. The supernatant of the first culture was transferred to the second culture in a new culture plate with (“5d” culture) or without (“3+2d” culture) immobilized mAbs to CD3, ICOS, and LFA-1. An equivalent volume of fresh medium containing IL-6 (20 ng/ml), TGF-β1 (1 ng/ml), anti-IL-2 (10 μg/ml), and anti-IL-4 (10 μg/ml) was added. (**B**) Naïve CD4^+^ T cells were cultured as in (A) (“3+2d” culture) except that culture supernatants of the first “3d” culture were removed and resuspended in twice the volume of fresh RPMI medium containing TGF-β1 (1 ng/ml), IL-6 (20 ng/ml), and mAbs to IL-2, CD25, and CD122 (10 μg/ml each), and the cells were further cultured in new culture plates for 2 days. CH-223191 (5 μM) was added in the first or second culture or both. (**A** and **B**) Data are presented as mean ± SD of triplicate samples. Results shown are representative of three independent experiments. (**C**) Naïve CD4^+^ T cells were cultured as in (A) (“3+2d” culture). CH-223191 (5 μM) or vehicle control was added in the first culture. The cells were assessed for chemotactic activity toward the chemokine CXCL13 (1 μg/ml) in transwells. Data are presented as mean ± SD of quadruplicate samples. Results shown are representative of five independent experiments. **p < 0.01, ***p < 0.001. ns, not significant.

CXCR5 expression slightly but significantly increased after the 2-step “3+2d” culture compared with that after the first “3d” culture or the “5d” culture in the RPMI medium (Fig 8A and S11 Fig in S1 File). Significant CH-223191-dependent upregulation of CXCR5 expression was observed after the “3+2d” culture, but not after the first “3d” culture or the “5d” culture (Fig 8A). The upregulation was observed only when CH-223191 was added in the first culture (Fig 8B). These results suggest that AhR activity prohibits the increase in CXCR5 expression after the release from TCR engagement, but that AhR-dependent signals during TCR engagement are responsible for the prohibition.

To test if the CXCR5 molecules expressed on Tfh-like cells generated in the presence or absence of CH-223191 in RPMI 1640 were functionally active, we performed transwell migration assays. The CH-223191-treated cells exhibited significant chemotaxis toward the CXCR5 ligand CXCL13 (Fig 8C), whereas FICZ-treated cells failed to enhance chemotaxis toward CXCL13 (S12A Fig in S1 File). Control cells tended to migrate toward CXCL13, although statistically not significant (Fig 8C and S12 Fig in S1 File). These results suggest that CXCR5 expressed on the CH-223191-treated Tfh-like cells was functionally active, and that regulation of the AhR activity affects their chemotactic capacity. We also confirmed that the CH-223191-treated cells could produce IL-21. Although CH-223191 at 5 μM moderately suppressed IL-21 production, CH-223191 at 2.5 μM suppressed neither IL-21 production nor *Bcl6* expression but significantly suppressed both *Rorc(γt)* expression and IL-17A production. It also enhanced chemotaxis toward CXCL13 (S12D Fig in S1 File).

CCR7 and CXCR4 are expressed on naïve T cells and downregulated during differentiation to effector T cells [54]. The expression levels of CCR7 and CXCR4 in the in vitro-generated cells were low and not affected by CH-223191 (S13 Fig in S1 File). Expression of CXCR4, but not CCR7, was downregulated after the “3+2d” culture compared with the “3d” culture. In sum, these results suggest that AhR activity regulates the differentiation balance between Tfh-like cells and Th17 cells.

### The CH-223191-treated Tfh-like cells do not express RORγt but exhibit BCL6 protein levels equivalent to Tfh cells in vivo

**Fig 9:**
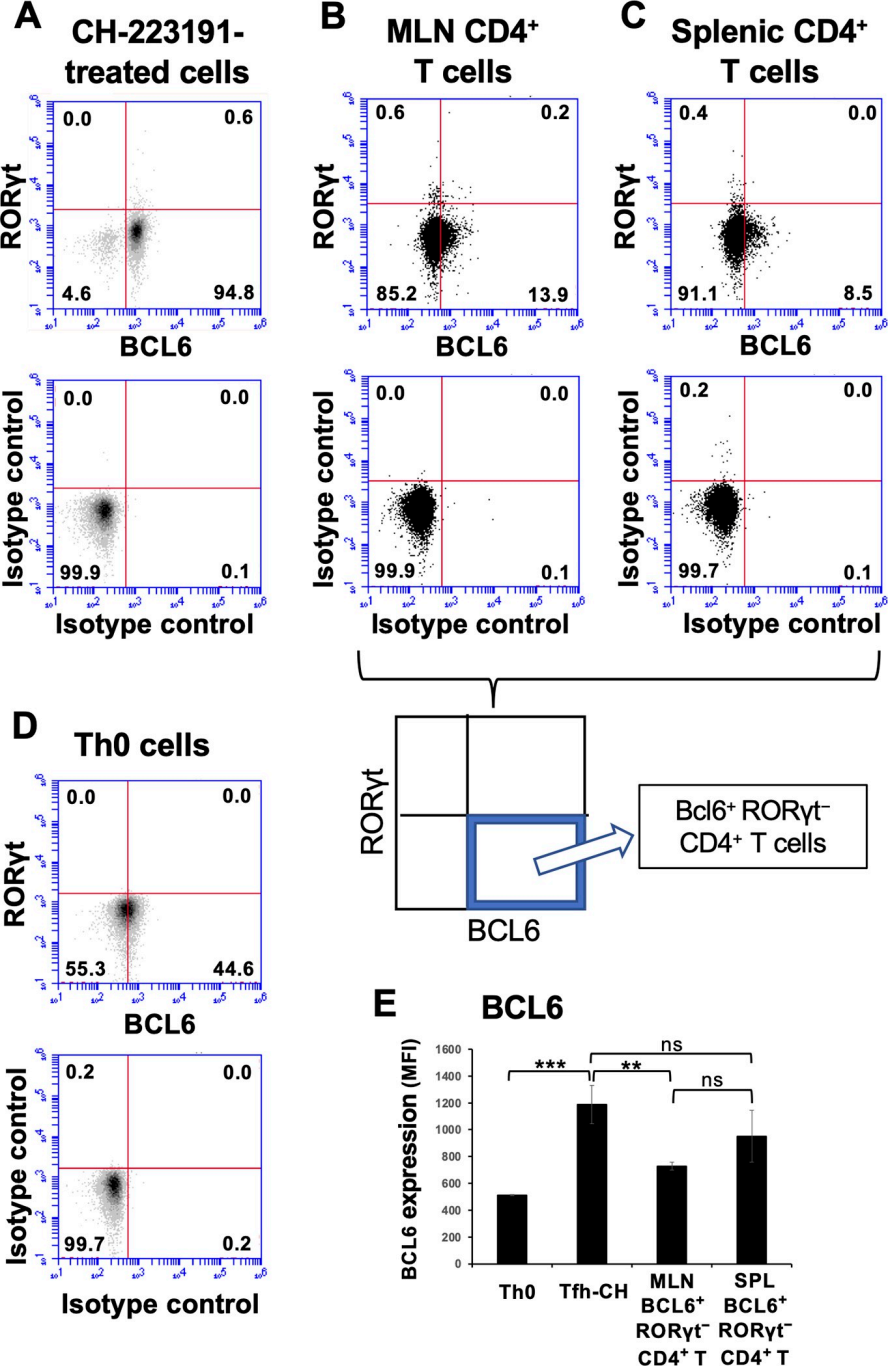
CH-223191-treated Tfh-like cells exhibit levels of BCL6 expression comparative to those observed in Tfh cells from immunized mice and negligible levels of RORγt expression. (**A**) CH-223191-treated Tfh-like cells were generated as described in the legend of Fig 8C. CH-223191 (5 μM) was added in the first culture. (**B** and **C**) CD4^+^ T cells were purified from mesenteric lymph nodes (MLN) and spleens (SPL) of OVA-immunized mice using a EasySep Mouse CD4^+^ T Cell Enrichment kit. (**D**) Th0 cells were generated in RPMI medium as described in Materials and Methods. Their expression of BCL6 and RORγt protein was determined by flow cytometry, and density plots (A and D) or dot plots (B and C) of their expression are shown. (**E**) BCL6 expression (MFI) levels in Th0, CH-2231-treated Tfh-like cells, and BCL6^+^ RORγt^−^ CD4^+^ T cells from MLN and SPL are compared. Data are presented as mean ± SD of three to seven samples. Results shown are representative of at least three independent experiments. *p < 0.05, **p < 0.01, ***p < 0.001. ns, not significant.

We examined protein expression of BCL6 and RORγt in CH-223191-treated Tfh-like cells. The most cells expressed BCL6 but did not express RORγt (Fig 9A). The expression levels of BCL6 and PD-1 were nearly equivalent to or even higher than those in Tfh cells from mesenteric lymph nodes and spleens of immunized mice (Fig 9B, Fig 9C, and S10 and S14 Figs in S1 File). However, the average expression levels of CXCR5 in CH-223191-treated Tfh-like cells appeared to be still lower than those in Tfh cells from immunized mice (S14E Fig in S1 File). These results suggest that Tfh-like cells generated at this stage well resemble Tfh cells in vivo but that additional factors may be required for higher expression of CXCR5.

## Discussion

To clarify the kinetic and molecular mechanisms of Tfh cell differentiation, we established an in vitro model system of mouse Tfh cell differentiation in the absence of other cell types. Upon TCR/CD3 stimulation of naïve CD4^+^ T cells in vitro, costimulation with immobilized mAbs to ICOS and LFA-1 and a soluble mAb to CD28 consistently led to enhanced expression of *Bcl6*, the master regulator gene for Tfh cell differentiation. ICOS signaling is essential for upregulating BCL6 expression through nuclear exclusion of FOXO1, and BCL6 upregulates Tfh marker molecules such as CXCR5, PD-1, and ICOS, and downregulates CCR7 [21, 24, 55]. LFA-1 stimulation also enhances BCL6 expression in a Talin-1-dependent manner [22]. BCL6 represses expression of microRNAs and Id2, which can suppress the expression of Tfh-specific genes and inhibit Tfh cell differentiation [6, 56, 57]. BCL6 also represses the development of other Th lineages by directly suppressing the promoter activities of the *Tbx21* and *Rorc* genes or by posttranscriptional regulation of *Gata3* in Tfh cells [4, 58]. BCL6 represses its own gene as well, while it indirectly induces the expression of several other genes by repressing repressors [9, 57]. The BCL6 and E3 ligase Cullin3 complex represses *Bcl6* and *Batf*, which are involved in activating the *Bcl6* promoter, and thus exerts negative feedback on the Tfh program [59]. Upregulation of *Bcl6* expression after the release from persistent TCR engagement in vitro might involve release from this negative feedback mechanism. Interestingly, the flow cytometric analysis of intracellular BCL6 protein revealed that the apparent BCL6 protein expression after the release from TCR engagement was downregulated unlike the Bcl6 mRNA expression. However, the cell sizes after the release, especially in the presence of added TGF-β1, appeared to be much smaller than those right after the TCR engagement, suggesting that BCL6 protein concentration might not be significantly downregulated after the release. Indeed, significant levels of BCL6 protein, as well as its mRNA expression, were detected in after the release from TCR engagement in the presence of TGF-β1. Alternatively, STAT activities might contribute to this phenomenon. TCR stimulation can induce delayed and prolonged activation of STAT5 as well as STAT3 [60]. In addition, persistent TCR activation may enhance IL-6-dependent production of IL-21, which can activate not only STAT3 but also STAT5. IL-6 and IL-21 induce BCL6 upregulation depending on STAT3 activation [10, 12], whereas STAT5 outcompetes STAT3 for binding to a regulatory region of the *Bcl6* gene and inhibits *Bcl6* expression [61].

TGF-β signals promote Tfh cell differentiation in human CD4^+^ T cells but their role in mouse Tfh cell differentiation has remained controversial [4, 7, 8, 36]. Our results suggest that TGF-β1 enhances the differentiation, proliferation, and survival of mouse Tfh-like cells in our culture system. The strict regulation of IL-2 signals with blocking Abs to both IL-2 and IL-2Rs might contribute to the TGF-β1-induced enhancement. We mainly used 1 ng/ml TGF-β1 in the present study. Higher concentrations (5 or 10 ng/ml) of TGF-β1 induced higher levels of *Bcl6* expression than 1 ng/ml TGF-β1. TGF-β suppresses IL-2Rα expression and STAT5 activation [36]. Accordingly, our preliminary experiments revealed that lower concentrations of anti-IL-2 and anti-IL-2R Abs were sufficient to enhance *Bcl6* expression in the presence of higher concentrations of TGF-β1. When blocking of IL-2 signaling was insufficient, the combination of TGF-β and residual IL-2 signals might induce regulatory T cells that suppress Tfh cell differentiation. However, we could not detect *Foxp3* expression under the present condition. The sensitivity of CD4^+^ T cells to IL-2 or TGF-β may differ between the two species under their culture conditions.

*Cxcr5* expression was not synchronously regulated with *Bcl6* expression. BCL6 is required for maintaining CXCR5 expression in T cells, but not for the expression in the early phase of the immune response [62]. We found that CXCR5 expression was enhanced after the release from persistent TCR engagement in the presence or absence of the AhR antagonist CH-223191. Expression of some other chemokine receptors including CXCR3 and CCR9, is also enhanced after the release from persistent TCR engagement in the presence of IFN-γ and retinoic acid, respectively [63, 64]. Higher doses of antigen and higher strength of TCR are considered to favor the induction of Tfh cell differentiation [65]. It may suggest that longer duration favors Tfh cell differentiation. However, medium-affinity TCRs bias mouse naïve T cells to become Tfh cells, while higher-affinity TCRs promote the formation of Th1 or Th17 cells [66]. Therefore, although persistent TCR engagement is likely to be required for Tfh cell differentiation, the optimal duration of TCR engagement for Tfh cell differentiation may vary depending on the nature and dose of the antigen and other factors.

AhR activity appeared to be critical for the differentiation balance between Tfh-like cells and Th17 cells. It is well known that AhR activity contributes to the Th17 cell differentiation [38–40], while its effects on Tfh cell differentiation varied depending on the agonist used in vivo [67]. A recent study in vivo indicated that AhR indeed modulates Tfh cell responses to influenza A virus infection in mice [68]. We found in our in vitro system that *Rorc(γt)* expression was upregulated by AhR agonists and downregulated by the antagonist, and that *Cxcr5* expression but not *Bcl6* expression was reciprocally regulated by these AhR modulators. Even by changing the medium from DMEM to RPMI 1640, which contains lower concentrations of aromatic amino acids, we observed the reciprocal regulation of *Rorc(γt)* and *Cxcr5* expression. The AhR activity during TCR engagement appeared to be responsible for suppressing CXCR5 expression after the release from TCR engagement. It remains unclear how AhR activity could be repressed in vivo. However, competition between AhR and other transcription factors such as hypoxia-inducible factor HIF-1α for binding to aryl hydrocarbon receptor nuclear translocator (ARNT) might contribute to the repression of AhR activity, since their transcriptional activities depend on the formation of heterodimers with ARNT [69]. HIF-1α depletion from CD4^+^ T cells reduces frequencies of antigen-specific GC B cells, Tfh cells [70].

Efficient induction of surface CXCR5 expression in mouse T cells in vitro has been difficult at least in the absence of antigen presenting cells [13, 14]. However, we could induce CXCR5-bearing Tfh-like cells that were capable of chemotaxis toward CXCL13. Similar levels of CXCR5 expression can be observed in a significant population of Tfh cells in vivo, but the mean CXCR5 expression levels in Tfh cells in vivo were significantly higher. Other factors may also contribute to the upregulation of CXCR5 expression in vivo. Furthermore, as Tfh cell differentiation appears to be a multi-step process and requires cognate interaction with B cells after the initial interaction with DCs [1, 9], the secondary TCR-mediated stimulation may be required to induce full maturation of the Tfh-like cells and higher CXCR5 expression in them in vitro.

The Tfh-like cells generated especially in DMEM without AhR antagonists might at least partially resemble Tfh17 cells, a subset of Tfh cells expressing both RORγt and BCL6 [71], although their expression of CXCR5 was low. Circulating Tfh (cTfh) cells, first found in human tonsils and blood, share the phenotype and functional nature of bona fide Tfh cells in GCs [71–73]. While Tfh cells as well as cTfh cells appear to be heterogenous in their phenotypes and functions reminiscent of conventional helper T cells, including Th1, Th2, and Th17 cells, the mechanism underlying their differentiation remains unclear [72, 73]. Multiple pathways are likely involved in the induction of Tfh cell subsets. In the differentiation of Tfh1-like cells, however, IL-12 signaling can be involved [15, 17]. Tfh17-like cells represent the most predominant subset in severe COVID-19 cases [74]. Regulation of the development of Tfh and cTfh cell subsets might change the disease outcome.

## Conclusion

We generated murine Tfh-like cells in vitro in the absence of other types of cells by stimulating naïve CD4^+^ T cells via CD3, ICOS, LFA-1, and CD28 in the presence of IL-6 and TGF-β, and blocking IL-2 signaling, followed by release from persistent TCR/CD3-mediated stimulation. These cells exhibited the fundamental key features of Tfh cells, including the expression of BCL6, PD-1, ICOS, and functional CXCR5 even if not high enough, and produced IL-21 and exhibited helper T cell activity. Suppression of AhR activity enhanced Tfh cell differentiation. The present culture system may provide a useful in vitro system for further analysis of the differentiation mechanisms of Tfh cells and Tfh subsets and for screening for modulators of their differentiation including CXCR5 expression.

## Supporting information

S1 File (PDF)

S1 Dataset Original FCM data for Figs and SFigs. (XLSX)

## References

[1] Crotty S. T follicular helper cell biology: A decade of discovery and diseases. Immunity. 2019; 50: 1132–1148. doi: 10.1016/j.immuni.2019.04.011 31117010 PMC6532429

[2] Shulman Z, Gitlin AD, Targ S, Jancovic M, Pasqual G, Nussenzweig MC, et al. T follicular helper cell dynamics in germinal centers Science. 2013; 341: 673–677. doi: 10.1126/science.1241680 23887872 PMC3941467

[3] Förster R, Mattis AE, Kremmer E, Wolf E, Brem G, Lipp M, A putative chemokine receptor, BLR1, directs B cell migration to defined lymphoid organs and specific anatomic compartments of the spleen. Cell. 1996; 87: 1037–1047. doi: 10.1016/s0092-8674(00)81798-5 8978608

[4] Nurieva RI, Chung Y, Martinez GJ, Yang XO, Tanaka S, Matskevitch TD, et al. Bcl6 mediates the development of T follicular helper cells. Science. 2009; 325: 1001–1005. https://www.science.org/doi/10.1126/science.1176676. 19628815 10.1126/science.1176676 PMC2857334

[5] Johnston RJ, Poholek AC, DiToro D, Yusuf I, Eto D, Barnett B, et al. Bcl6 and Blimp-1 are reciprocal and antagonistic regulators of T follicular helper cell differentiation. Science. 2009; 325: 1006–1010. https://www.science.org/doi/10.1126/science.1175870. 19608860 10.1126/science.1175870 PMC2766560

[6] Yu D, Rao S, Tsai LM, Lee SK, He Y, Sutcliffe EL, et al. The transcriptional repressor Bcl-6 directs T follicular helper cell lineage commitment. Immunity. 2009; 31: 457–468. doi: 10.1016/j.immuni.2009.07.002 19631565

[7] Schmitt N, Liu Y, Bentebibel SE, Munagala I, Bourdery L, Venuprasad K, et al. The cytokine TGF-β co-opts signaling via STAT3-STAT4 to promote the differentiation of human TFH cells. Nat Immunol. 2014; 15: 856–865. https://www.nature.com/articles/ni.2947. 25064073 10.1038/ni.2947 PMC4183221

[8] Locci M, Wu JE, Arumemi F, Mikulski Z, Dahlberg C, Miller AT, et al. Activin A programs the differentiation of human TFH cells. Nat Immunol. 2016; 17: 976–984. https://www.nature.com/articles/ni.3494. doi: 10.1038/ni.3494 27376469 PMC4955732

[9] Choi J, Crotty S, Bcl6-mediated transcriptional regulation of follicular helper T cells (TFH). Trends Immunol. 2021; 42: 336–349. doi: 10.1016/j.it.2021.02.002 33663954 PMC8021443

[10] Nurieva RI, Chung Y, Hwang D, Yang XO, Kang HS, Ma L, et al. Generation of T follicular helper cells is mediated by interleukin-21 but independent of T helper 1, 2, or 17 cell lineages. Immunity. 2008; 29: 138–149. 10.1016/j.immuni.2008.05.009. 18599325 PMC2556461

[11] Eddahri F, Denanglaire S, Bureau F, Spolski R, Leonard WJ, Leo O, et al. Interleukin-6/STAT3 signaling regulates the ability of naive T cells to acquire B-cell help capacities. Blood. 2009; 113: 2426–2433. doi: 10.1182/blood-2008-04-154682 19020307 PMC2656270

[12] Eto D, Lao C, DiToro D, Barnett B, Escobar TC, Kageyama R, et al. IL-21 and IL-6 are critical for different aspects of B cell immunity and redundantly induce optimal follicular helper CD4 T cell (Tfh) differentiation. PLoS ONE. 2011; 6: e17739. doi: 10.1371/journal.pone.0017739 21423809 PMC3056724

[13] Lu KT, Kanno Y, Cannons JL, Handon R, Bible P, Elkahloun AG, et al. Functional and epigenetic studies reveal multistep differentiation and plasticity of In vitro-generated and in vivo-derived follicular T helper cells. Immunity. 2011; 35: 622–632. 10.1016/j.immuni.2011.07.015. 22018472 PMC3235706

[14] Nakayamada S, Kanno Y, Takahashi H, Jankovic D, Lu KT, Johnson TA, et al. Early Th1 cell differentiation is marked by a Tfh cell-like transition. Immunity. 2011; 35: 919–931. doi: 10.1016/j.immuni.2011.11.012 22195747 PMC3244883

[15] Oestreich KJ, Mohn SE, Weinmann AS. Molecular mechanisms that control the expression and activity of Bcl-6 in T_H_1 cells to regulate flexibility with a T_FH_-like gene profile. Nat Immunol. 2012; 13: 405–411. https://www.nature.com/articles/ni.2242. 22406686 10.1038/ni.2242 PMC3561768

[16] Zhu Y, Zhao Y, Zou L, Zhang D, Aki D, Liu YC. The E3 ligase VHL promotes follicular helper T cell differentiation via glycolytic-epigenetic control. J Exp Med. 2019; 216: 1664–1681. doi: 10.1084/jem.20190337 31123085 PMC6605754

[17] Powell MD, Read KA, Sreekumar BK, Jones DM, Oestreich KJ. IL-12 signaling drives the differentiation and function of a TH1-derived TFH1-like cell population. Sci Rep. 2019; 9: 13991. https://www.nature.com/articles/s41598-019-50614-1 doi: 10.1038/s41598-019-50614-1 31570752 PMC6769002

[18] Gao X, Wang H, Chen Z, Zhou P, Yu D. An optimized method to differentiate mouse follicular helper T cells in vitro. Cell Mol Immunol. 2020; 17: 779–781. doi: 10.1038/s41423-019-0329-7 31754234 PMC7331742

[19] Badell IR, La Muraglia GM II, Liu D, Wagener ME, Ding G, Ford MI. Selective CD28 blockade results in superior inhibition of donor-specific T follicular helper cell and antibody responses relative to CTLA4-Ig. Am J Transplant. 2018; 18: 89–101. doi: 10.1111/ajt.14400 28637095 PMC5740006

[20] Akiba H, Takeda K, Kojima Y, Usui Y, Harada N, Yamazaki T, et al. The role of ICOS in the CXCR5^+^ follicular B helper T cell maintenance in vivo. J Immunol. 2005; 175: 2340–2348. 10.4049/jimmunol.175.4.2340. 16081804

[21] Choi YS, Kageyama R, Eto D, Escobar TC, Johnston RJ, Monticelli L, et al. ICOS receptor instructs T follicular helper cell versus effector cell differentiation via induction of the transcriptional repressor Bcl6. Immunity. 2011; 34: 932–946. doi: 10.1016/j.immuni.2011.03.023 21636296 PMC3124577

[22] Meli AP, Fontés G, Avery DT, Leddon SA, Tam M, Elliot M, et al. The integrin LFA-1 controls T follicular helper cell generation and maintenance. Immunity. 2016; 45: 831–846. doi: 10.1016/j.immuni.2016.09.018 27760339 PMC5672956

[23] Tahiliani V, Hutchinson TE, Abboud G, Croft M, Salek-Ardakani S. OX40 cooperates with ICOS to amplify follicular Th cell development and germinal center reactions during infection. J Immunol. 2017; 198: 218–228. doi: 10.4049/jimmunol.1601356 27895177 PMC5173420

[24] Wan S, Ni L, Zhao X, Liu X, Xu W, Jin W, et al. Costimulation molecules differentially regulate the ERK-Zfp831 axis to shape T follicular helper cell differentiation. Immunity. 2021; 54: 2740–2755. doi: 10.1016/j.immuni.2021.09.018 34644536

[25] Vogelzang A, McGuire HM, Yu D, Sprent J, Mackay CR, King C. A fundamental role for interleukin-21 in the generation of T follicular helper cells. Immunity. 2008; 29: 127–137. doi: 10.1016/j.immuni.2008.06.001 18602282

[26] Zhou L, Ivanov II, Spolski R, Min R, Shenderov K, Egawa T, et al. IL-6 programs T_H_-17 cell differentiation by promoting sequential engagement of the IL-21 and IL-23 pathways. Nat Immunol. 2007; 8: 967–974. https://www.nature.com/articles/ni1488. 17581537 10.1038/ni1488

[27] Nurieva R, Yang XO, Martinez G, Zhang Y, Panopoulos AD, Ma L, et al. Essential autocrine regulation by IL-21 in the generation of inflammatory T cells. Nature. 2007; 448: 480–483. https://www.nature.com/articles/nature05969. doi: 10.1038/nature05969 17581589

[28] Suto A, Kashiwakuma D, Kagami S, Hirose K, Watanabe N, Yokote K, et al. Development and characterization of IL-21-producing CD4^+^ T cells. J Exp Med. 2008; 205: 1369–1379. 10.1084/jem.20072057. 18474630 PMC2413034

[29] Schmitt N, Morita R, Bourdery L, Bentebibel SE, Zurawski SM, Banchereau J, et al. Human dendritic cells induce the differentiation of interleukin-21-producing T follicular helper-like cells through interleukin-12. Immunity. 2009; 31: 158–169. doi: 10.1016/j.immuni.2009.04.016 19592276 PMC2731623

[30] Ma CS, Suryani S, Avery DT, Chan A, Nanan R, Santner-Nanan B, E. et al. Early commitment of naïve human CD4+ T cells to the T follicular helper (TFH) cell lineage is induced by IL-12, Immunol Cell Biol. 2009; 87: 590–600. 10.1038/icb.2009.64. 19721453

[31] Ballesteros-Tato A, León B, Graf BA, Moquin A, Adams PS, Lund FE, et al. Interleukin-2 inhibits germinal center formation by limiting T follicular helper cell differentiation. Immunity. 2012; 36: 847–856. doi: 10.1016/j.immuni.2012.02.012 22464171 PMC3361521

[32] Johnston RJ, Choi YS, Diamond JA, Yang JA, Crotty S. STAT5 is a potent negative regulator of T_FH_ cell differentiation. J Exp Med. 2012; 209: 243–250. 10.1084/jem.20111174. 22271576 PMC3281266

[33] Liao W, Lin JX, Wang L, Li P, Leonard WJ. Modulation of cytokine receptors by IL-2 broadly regulates differentiation into helper T cell lineages. Nat Immunol. 2011; 12: 551–559. https://www.nature.com/articles/ni.2030. doi: 10.1038/ni.2030 21516110 PMC3304099

[34] DiToro D, Winstead CJ, Pham D, Witte S, Andargachew R, Singer JR, et al. Differential IL-2 expression defines developmental fates of follicular versus nonfollicular helper T cells. Science. 2018; 361: eaao2933. https://www.science.org/doi/ doi: 10.1126/science.aao2933 30213884 PMC6501592

[35] Papillion A, Powell MD, Chisolm DA, Bachus H, Fuller MJ, Weinmann AS, et al. Inhibition of IL-2 responsiveness by IL-6 is required for the generation of GC-TFH cells. Sci Immunol. 2019; 4: eaaw7636. https://www.science.org/doi/10.1126/sciimmunol.aaw7636. 31519812 10.1126/sciimmunol.aaw7636 PMC6820141

[36] Marshall HD, Ray JP, Laidlaw BJ, Zhang N, Gawande D, Staron MM, et al. The transforming growth factor beta signaling pathway is critical for the formation of CD4 T follicular helper cells and isotype-switched antibody responses in the lung mucosa. eLife. 2015; 4: e04851. https://elifesciences.org/articles/04851. doi: 10.7554/eLife.04851 25569154 PMC4337607

[37] Wei L, Laurence A, Elia KM, O’Shea JJ. IL-21 is produced by Th17 cells and drives IL-17 production in a STAT3-dependent manner. J Biol Chem. 2007; 282: 34605–34610. doi: 10.1074/jbc.M705100200 17884812 PMC2323680

[38] Quintana FJ, Basso AS, Iglesias AH, Korn T, Farez MF, Bettelli E, et al. Control of T_reg_ and T_H_17 cell differentiation by the aryl hydrocarbon receptor. Nature. 2008; 453: 65–71. https://www.nature.com/articles/nature06880. 18362915 10.1038/nature06880

[39] Veldhoen M, Hirota K, Westendorf AM, Buer J, Dumoutier L, Renauld JC, et al. The aryl hydrocarbon receptor links T_H_17-cell-mediated autoimmunity to environmental toxins. Nature. 2008; 453: 106–109. https://www.nature.com/articles/nature06881. 18362914 10.1038/nature06881

[40] Kimura A, Naka T, Nohara K, Fujii-Kuriyama Y, Kishimoto T. Aryl hydrocarbon receptor regulates Stat1 activation and participates in the development of Th17 cells. Proc Natl Acad Sci USA. 2008; 105: 9721–9726. doi: 10.1073/pnas.0804231105 18607004 PMC2474493

[41] Öberg M, Bergander L, Hakansson H, Rannug U, Rannug A. Identification of the tryptophan photoproduct 6-formylindolo[3,2-*b*]carbazole, in cell culture medium, as a factor that controls the background aryl hydrocarbon receptor activity. Toxicol Sci. 2005; 85: 935–943. 10.1093/toxsci/kfi154. 15788723

[42] Veldhoen M, Hirota K, Christensen J, O’Garra A, Stockinger B. Natural agonists for aryl hydrocarbon receptor in culture medium are essential for optimal differentiation of Th17 T cells. J Exp Med. 2009; 206: 43–49. doi: 10.1084/jem.20081438 19114668 PMC2626686

[43] Prigent L, Robineau M, Jouneau S, Morzadec C, Louarn L, Vernhet L, et al. The aryl hydrocarbon receptor is functionally upregulated early in the course of human T-cell activation. Eur J Immunol. 2014; 44: 1330–1340. doi: 10.1002/eji.201343920 24549985

[44] Yokota-Nakatsuma A, Takeuchi H, Ohoka Y, Kato C, Song SY, Hoshino T, et al. Retinoic acid prevents mesenteric lymph node dendritic cells from inducing IL-13-producing inflammatory Th2 cells, Muc Immunol. 2014; 7: 786–801. https://www.nature.com/articles/mi201396. doi: 10.1038/mi.2013.96 24220301

[45] Quah BJC, Parish CR. The use of carboxyfluorescein diacetate succinimidyl ester (CFSE) to monitor lymphocyte proliferation. J Vis Exp. 2010; 44: e2259. https://www.jove.com/video/2259. doi: 10.3791/2259 20972413 PMC3185625

[46] Iwata M, Hirakiyama A, Eshima Y, Kagechika H, Kato C, Song SY. Retinoic acid imprints gut-homing specificity on T cells, Immunity 2004; 21: 527–538. doi: 10.1016/j.immuni.2004.08.011 15485630

[47] Dong L, He Y, Zhou S, Cao Y, Li Y, Bi Y, et al. HIF1α-dependent metabolic signals control the differentiation of follicular helper T cells. Cells 2019; 8: 1450. 10.3390/cells8111450. 31744227 PMC6912655

[48] Veldhoen M, Hocking RJ, Atkins CJ, Locksley RM, Stockinger B. TGFβ in the context of an inflammatory cytokine milieu supports de novo differentiation of IL-17-producing T cells. Immunity. 2006; 24: 179–189. 10.1016/j.immuni.2006.01.001. 16473830

[49] Wurster AL, Rodgers VL, Satoskar AR, Whitters MJ, Young DA, Collins M, et al. Interleukin 21 is a T helper (Th) cell 2 cytokine that specifically inhibits the differentiation of naive Th cells into interferon γ–producing Th1 cells. J Exp Med. 2002; 19: 969–977. 10.1084/jem.20020620. PMC2194031 12370258

[50] Korn T, Bettelli E, Gao W, Awasthi A, Jäger A, Strom TB, et al. IL-21 initiates an alternative pathway to induce proinflammatory T_H_17 cells. Nature 448 (2007) 484–487. https://www.nature.com/articles/nature05970. 17581588 10.1038/nature05970 PMC3805028

[51] Kim SH, Henry EC, Kim DK, Kim YH, Shin KJ, Han MS, et al. Novel compound 2-methyl-2H-pyrazole-3-carboxylic acid (2-methyl-4-o-tolylazo-phenyl)-amide (CH-223191) prevents 2,3,7,8-TCDD-induced toxicity by antagonizing the aryl hydrocarbon receptor. Mol Pharmacol. 2006; 69: 1871–1878. doi: 10.1124/mol.105.021832 16540597

[52] Rannug U, Rannug A, Sjöberg U, Li H, Westerholm R, Bergman J. Structure elucidation of two tryptophan-derived, high affinity Ah receptor ligands. Chem Biol. 1995; 2: 841–845. doi: 10.1016/1074-5521(95)90090-x 8807817

[53] Song J, Clagett-Dame M, Peterson RE, Hahn ME, Westler WM, Sicinski RR, et al. A ligand for the aryl hydrocarbon receptor isolated from lung. Proc Natl Acad Sci USA. 2002; 99: 14694–14699. doi: 10.1073/pnas.232562899 12409613 PMC137481

[54] Kim CH, Nagata K, Butcher EC. Dendritic cells support sequential reprogramming of chemoattractant receptor profiles during naive to effector T cell differentiation. J Immunol. 2003; 171: 152–158. doi: 10.4049/jimmunol.171.1.152 12816993

[55] Panneton V, Chang J, Witalis M, Li J, Suh WK. Inducible T-cell co-stimulator: Signaling mechanisms in T follicular helper cells and beyond. Immunol Rev. 2019; 291: 91–103. doi: 10.1111/imr.12771 31402504

[56] Shaw LA, Bélanger S, Omilusik KD, Cho S, Scott-Browne JP, Nance JP, et al. Id2 reinforces T_H_1 differentiation and inhibits E2A to repress T_FH_ differentiation. Nat Immunol. 2016; 17: 834–843. https://www.nature.com/articles/ni.3461. 27213691 10.1038/ni.3461 PMC4915968

[57] Choi J, Diao H, Faliti CE, Truong J, Rossi M, Bélanger S, et al. Bcl-6 is the nexus transcription factor of T follicular helper cells (TFH) via repressor-of-repressor circuits. Nat Immunol. 2020; 21: 777–789. https://www.nature.com/articles/s41590-020-0706-5. 32572238 10.1038/s41590-020-0706-5 PMC7449381

[58] Kusam S, Toney LM, Sato H, Dent AL. Inhibition of Th2 differentiation and GATA-3 expression by BCL-6. J Immunol. 2003; 170: 2435–2441. doi: 10.4049/jimmunol.170.5.2435 12594267

[59] Mathew R, Mao A, Chiang AH, Bertozzi-Villa C, Bunker JJ, Scanlon ST, et al. A negative feedback loop mediated by the Bcl6–cullin 3 complex limits Tfh cell differentiation. J Exp Med. 2014; 211: 1137–1151. doi: 10.1084/jem.20132267 24863065 PMC4042651

[60] Chueh FY, Yu CL. Engagement of T-cell antigen receptor and CD4/CD8 co-receptors induces prolonged STAT activation through autocrine/paracrine stimulation in human primary T cells. Biochem Biophys Res Commun. 2012; 426: 242–246. doi: 10.1016/j.bbrc.2012.08.074 22935418 PMC3597241

[61] Walker SR, Nelson EA, Yeh JE, Pinello L, Yuan GC, Frank DA. STAT5 outcompetes STAT3 to regulate the expression of the oncogenic transcriptional modulator BCL6. Mol Cell Biol. 2013; 33: 2879–2890. doi: 10.1128/MCB.01620-12 23716595 PMC3719667

[62] Liu X, Yan X, Zhong B, Nurieva RI, Wang A, Wang X, et al. Bcl6 expression specifies the T follicular helper cell program in vivo. J Exp Med. 2012; 209: 1841–1852. doi: 10.1084/jem.20120219 22987803 PMC3457730

[63] Nakajima C, Mukai T, Yamaguchi N, Morimoto Y, Park WR, Iwasaki M, et al. Induction of the chemokine receptor CXCR3 on TCR-stimulated T cells: dependence on the release from persistent TCR-triggering and requirement for IFN-γ stimulation. Eur J Immunol. 2002; 32: 1792–1801. 10.1002/1521-4141(200206)32:6<1792::AID-IMMU1792>3.0.CO;2-0. 12115663

[64] Ohoka Y, Yokota A, Takeuchi H, Maeda N, Iwata M. Retinoic acid-induced CCR9 expression requires transient TCR stimulation and cooperativity between NFATc2 and the retinoic acid receptor/retinoid X receptor complex. J Immunol. 2011; 186: 733–744. doi: 10.4049/jimmunol.1000913 21148038

[65] Baumjohan D, Fazilleau N. Antigen-dependent multistep differentiation of T-follicular helper cells and its role in SARS-CoV-2 infection and vaccination. Eur J Immunol. 2021; 51: 1325–1333. 10.1002/eji.202049148 33788271 PMC8250352

[66] Kotov DI, Mitchell JS, Pengo T, Ruedl C, Way SS, Langlois RA, et al. TCR Affinity Biases Th Cell Differentiation by Regulating CD25, Eef1e1, and Gbp2. J Immunol. 2019; 202: 2535–2545. https://www.jimmunol.org/content/202/9/2535. doi: 10.4049/jimmunol.1801609 30858199 PMC6478541

[67] Boule LA, Burke CG, Jin GB, Lawrence BP. Aryl hydrocarbon receptor signaling modulates antiviral immune responses: ligand metabolism rather than chemical source is the stronger predictor of outcome. Sci Rep. 2018; 8: 1826. https://www.nature.com/articles/s41598-018-20197-4. doi: 10.1038/s41598-018-20197-4 29379138 PMC5789012

[68] Houser CL, Lawrence BP. The aryl hydrocarbon receptor modulates T follicular helper cell responses to influenza virus infection in mice. J Immunol. 2022; 208: 2319–2330. https://www.jimmunol.org/content/208/10/2319. doi: 10.4049/jimmunol.2100936 35444027 PMC9117429

[69] Vorrink SU, Domann FE. Regulatory crosstalk and interference between the xenobiotic and hypoxia sensing pathways at the AhR-ARNT-HIF1α signaling node. Chem Biol Interact. 2014; 218: 82–88. 10.1016/j.cbi.2014.05.001. 24824450 PMC4091760

[70] Cho SH, Raybuck AL, Blagih J, Kemboi E, Haase VH, Jones RG, et al. Hypoxia-inducible factors in CD4+ T cells promote metabolism, switch cytokine secretion, and T cell help in humoral immunity. Proc Natl Acad Sci USA. 2019; 116: 8975–8984. https://www.pnas.org/cgi/doi/10.1073/pnas.1811702116 30988188 10.1073/pnas.1811702116 PMC6500120

[71] Hirota K, Turner JE, Villa M, Duarte JH, Demengeot J, Steinmetz OM, et al. Plasticity of Th17 cells in Peyer’s patches is responsible for the induction of T cell-dependent IgA responses. Nat Immunol. 2013; 14: 372–379. https://www.nature.com/articles/ni.2552. doi: 10.1038/ni.2552 23475182 PMC3672955

[72] Ueno H. Human circulating T follicular helper cell subsets in health and disease. J Clin Immunol. 2016; 36: 34–39. doi: 10.1007/s10875-016-0268-3 26984851

[73] Olatunde AC, Hale JS, Lamb TJ. Cytokine-skewed Tfh cells: functional consequences for B cell help. Trends Immunol. 2021; 42: 536–550. doi: 10.1016/j.it.2021.04.006 33972167 PMC9107098

[74] Golovki A, Kalinina O, Bezrukikh V, Aquino A, Zaikova E, Karonova T, et al. Imbalanced immune response of T-cell and B-cell subsets in patients with moderate and severe COVID-19. Viruses. 2021; 13: 1966. doi: 10.3390/v13101966 34696395 PMC8538447

