# Supplementary material for: Release from persistent T cell receptor engagement and blockade of aryl hydrocarbon receptor activity enhance IL-6-dependent mouse follicular helper T-like cell differentiation in vitro
Source: PLoS One. 2023 Jun 23;18(6):e0287746. doi: 10.1371/journal.pone.0287746 (PMC10289413; doi:10.1371/journal.pone.0287746)
Supplement: S1 File — (PDF) [file pone.0287746.s001.pdf]

S1 Table. Sequences of the primers for real-time PCR

Primers		Primer Sequence (5′-3′)
Rplp0	Forward	GGTGCCACACTCCATCATCA
	Reverse	CGCAAATGCAGATGGATCAG
Bcl6	Forward	TCATCAAGGCCAGTGAAGCA
	Reverse	ATTGTTCTCCACGACCTCACG
Rorc(γt)	Forward	CCGCTGAGAGGGCTTCAC
	Reverse	TGCAGGAGTAGGCCACATTACA
Prdm1	Forward	GTTCCCAAGAATGCCAACAG
	Reverse	GCATCCAGTTGCTTTTCTCC
Tbx21	Forward	TCAACCAGCACCAGACAGAG
	Reverse	AAACATCCTGTAATGGCTTGTG
Gata3	Forward	CTTATCAAGCCCAAGCGAAG
	Reverse	CCCATTAGCGTTCCTCCTC
Cxcr5	Forward	CTCGACATCAGACAGTGACCA
	Reverse	ACTGTAGAAGGCCAGTTCCTTG
Ahr	Forward	TGATGCCAAAGGGCAGCTTA
	Reverse	CATTGGACTGGACCCACCTC
Cyp1a1	Forward	TGGTCGTGTCAGTAGCCAAT
	Reverse	GAGTTAGGCAGGTAACGGAGG

S2 Table. Comparison of the aromatic amino acid content in DMEM and RPMI 1640 (mg/L)

Aromatic amino acid	DMEM	RPMI 1640
L-Tryptophan	16	5
L-Phenylalanine	66	15
L-Tyrosine 2Na $2H_2O$	104	29
L-Histidine	42	15

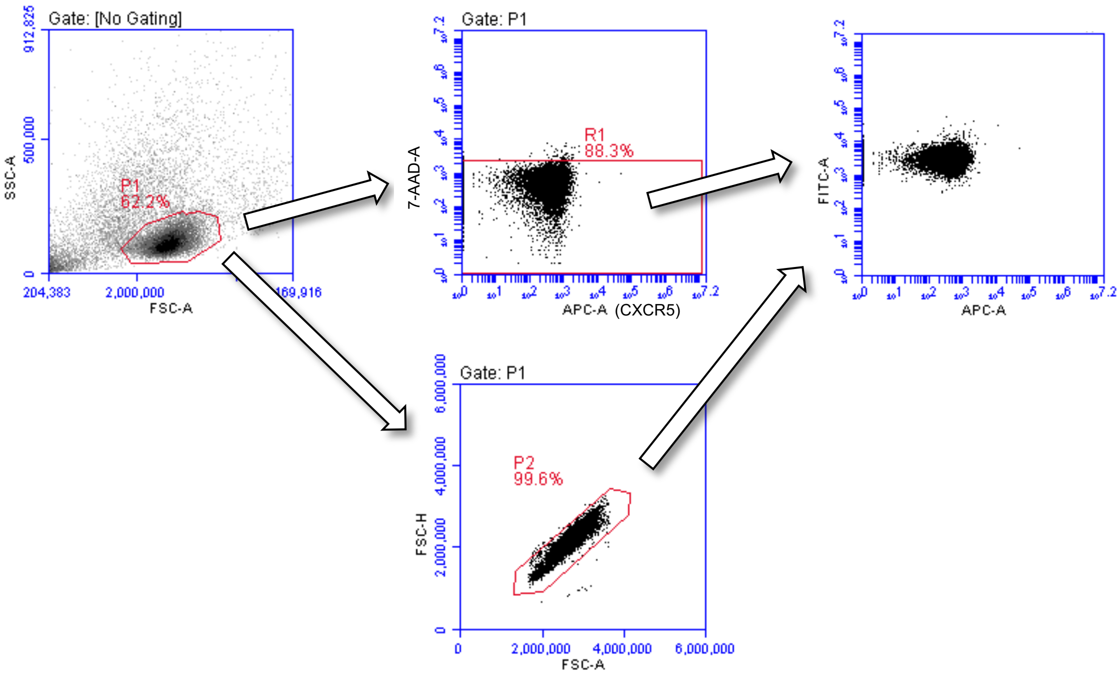

S1 Fig. Typical gating strategies for flow cytometric analysis of cultured cells. Live cells in cultured $CD4^+$ T cells were gated with FSC-A and SSC-A, followed by excluding 7-AAD-labeled cells from the gated cells. Doublets were excluded by using the FSC-A/FSC-H gating.

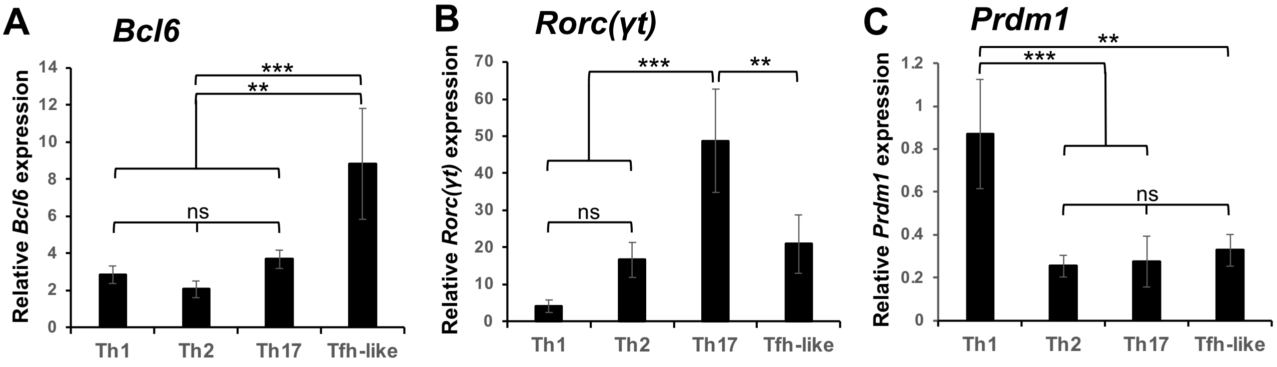

S2 Fig. Expression of *Bcl6*, *Rorc(γt)*, and *Prdm1* in CD4^{+} T cells cultured for 3 days under the Th1, Th2, Th17, and Tfh-like cell conditions. The "Th1", "Th2", and "Th17" cells were induced as described in the Materials and Methods but by culturing only for the first 3 days. "Tfh-like" cells were induced in the presence of both anti-IL-2 and anti-IL-2Rs as described in the legend of Fig 1. Relative mRNA expression of (**A**) *Bcl6*, (**B**) *Rorc(γt)*, and (**C**) *Prdm1* was analyzed by real-time PCR. Data are presented as mean ± SD of triplicate or quadruplicate samples. Results shown are representative of two independent experiments. ***$p < 0.001$, **$p < 0.01$. ns, not significant.

A

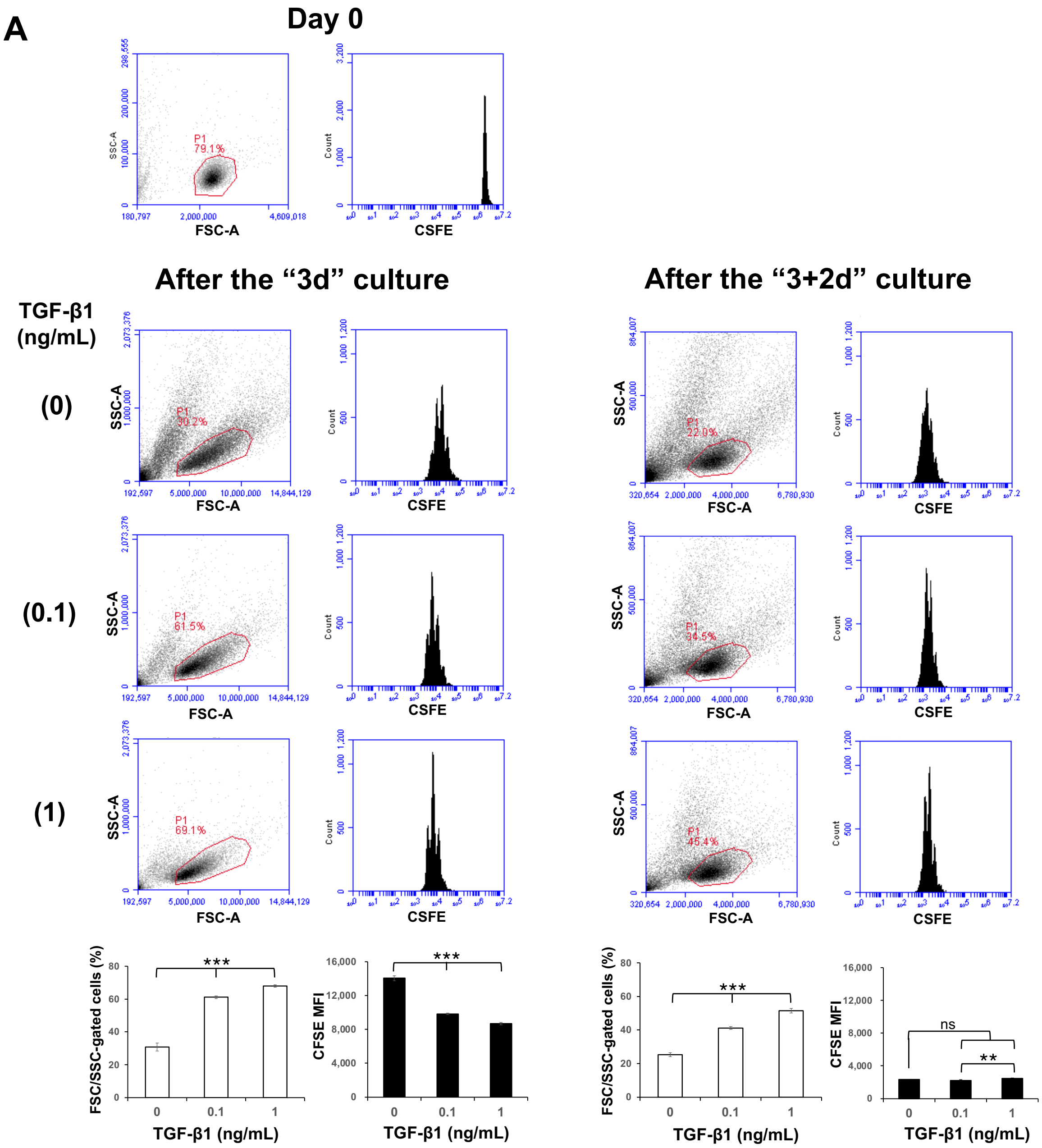

B

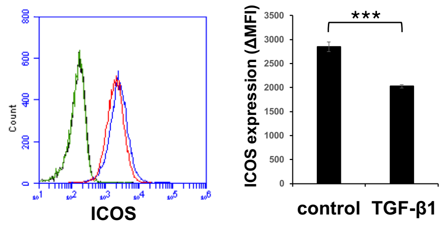

S3 Fig. Effects of TGF-β1 on CD4$^+$ T cell proliferation, survival, and ICOS expression. (**A**) Naive CD4$^+$ T cells were labeled with CFSE and cultured under the Tfh-like cell-inducing conditions as in Fig 3, with or without adding TGF-β1 (0.1 or 1 ng/mL). Live cells were gated based on the FSC/SSC. CFSE fluorescence intensity was analyzed by flow cytometry. MFI ± SD of quadruplicate samples were presented. $^{**}p < 0.01$, $^{***}p < 0.001$. ns, not significant. (**B**) ICOS expression in Tfh-like cells induced by the "3+2d" culture with or without adding TGF-β1 (1 ng/mL) was analyzed by flow cytometry (blue, TGF-β(-); red, TGF-β(+); black and green, isotype controls of TGF-β(-) and TGF-β(+), respectively). ΔMFI ± SD of triplicate samples were presented. $^{***}p < 0.001$. Results shown are representative of two or three independent experiments.

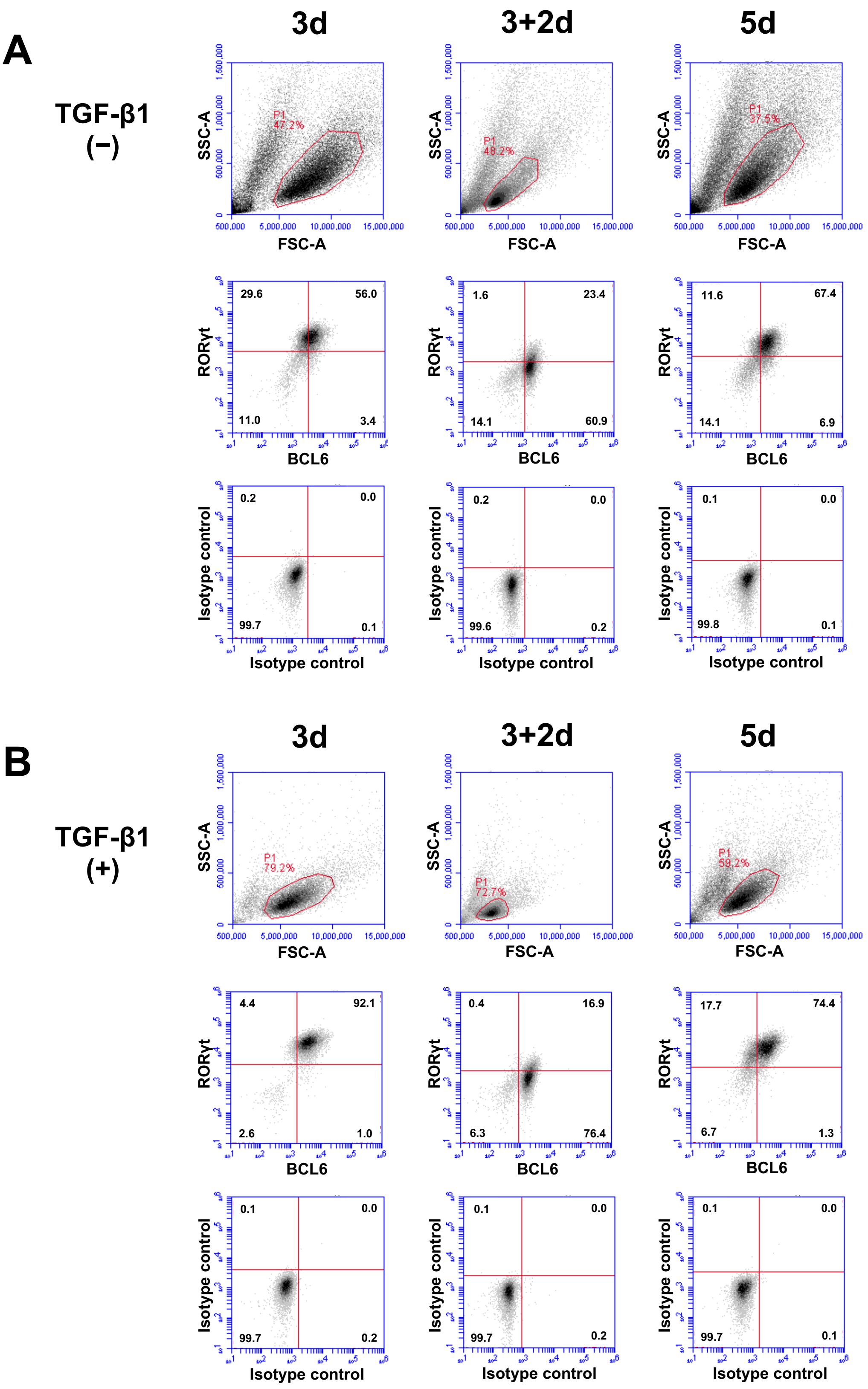

S4 Fig. Representative flow cytometry dot plots and density plots for Fig 4. Naïve CD4$^+$ T cells were cultured in the presence or absence of added TGF-β1 (1 ng/mL) as described in the legend of Fig 4. Briefly, the cells after the “3d” culture, “3+2d” culture, or “5d” culture were analyzed for FSC-A intensities. Aliquots of the cells were fixed and permeabilized, and analyzed for the intracellular expression of BCL6 and RORγt protein. BCL6 and RORγt expression levels are shown as density plots. Results shown are representative of three independent experiments.

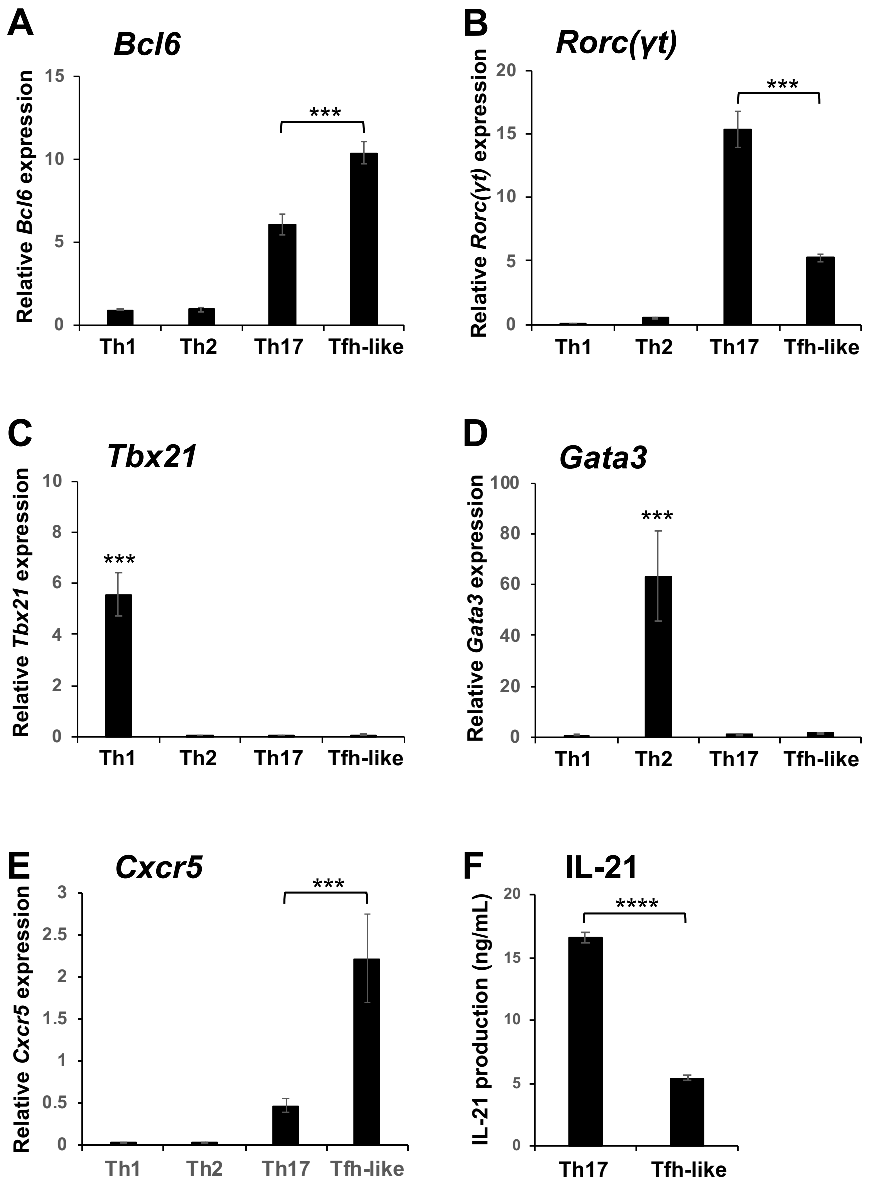

S5 Fig. The Tfh-like cells are distinct from Th17, Th1, and Th2 cells. Th1, Th2, and Th17 cells were induced from native $CD4^+$ T cells in vitro as described in the Materials and Methods. Tfh-like cells were induced by the "3+2d" culture in the presence of 1 ng/ml TGF-β1 as described in the legend of Fig 3. Relative mRNA expression of (**A**) *Bcl6*, (**B**) *Rorc(γt)*, (**C**) *Tbx21*, and (**D**) *Gata3* was analyzed by real-time PCR. Data are presented as mean ± SD of triplicate samples. (**E**) Th17 cells and Tfh-like cells were restimulated with immobilized mAbs to CD3 and CD28. IL-21 concentrations in the supernatant were assessed by ELISA. Data are presented as mean ± SD of quadruplicate samples. Results shown are representative of two independent experiments. ***$p < 0.001$, ****$p < 0.0001$.

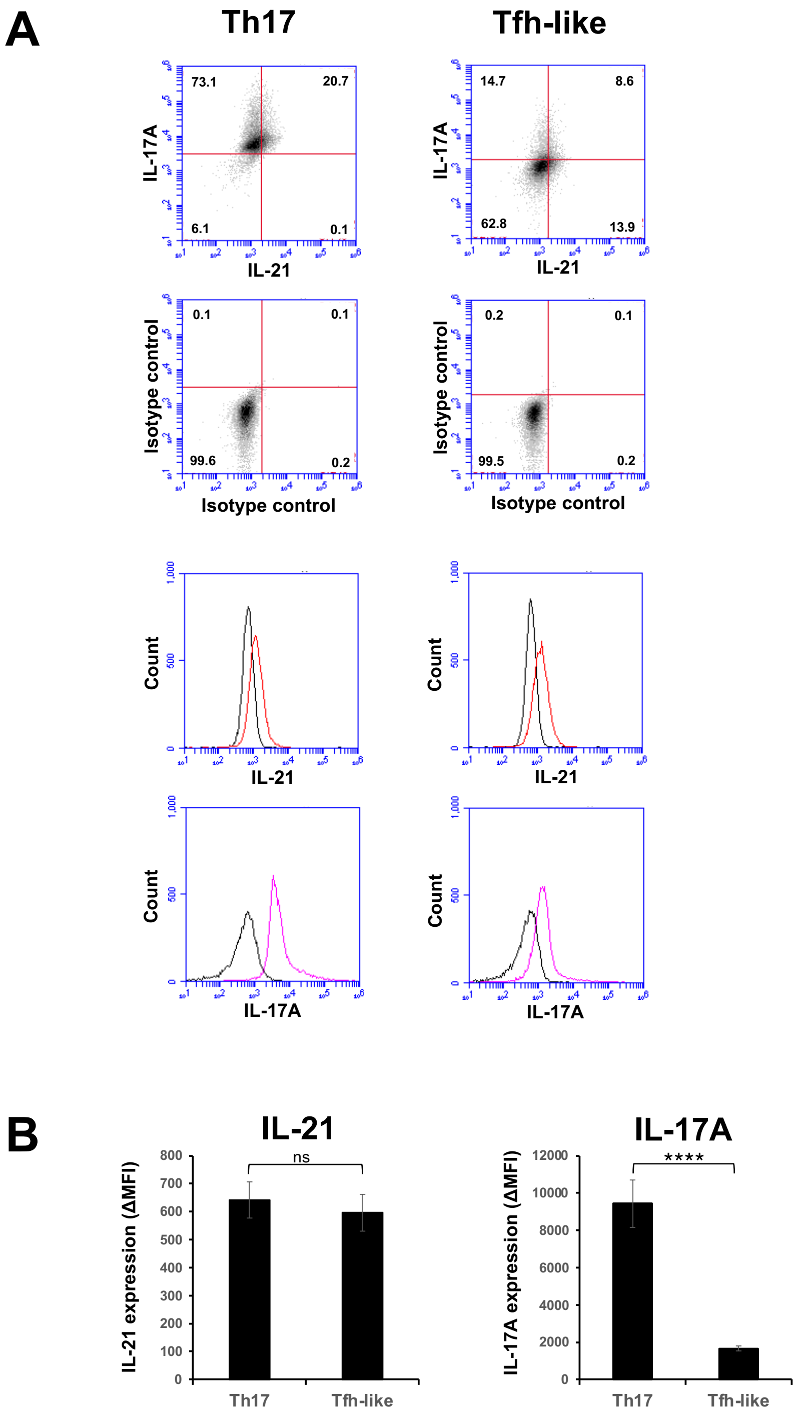

S6 Fig. The Tfh-like cells produce IL-21 at similar levels to Th17 cells but produce IL-17A at much lower levels than Th17 cells. Th17 cells and Tfh like cells were restimulated with immobilized mAbs to CD3 and CD28 for 2 days, and monensin was added for the last 2 hours. The cells were fixed and permeabilized, and their intracellular IL-21 and IL-17A expression was analyzed by flow cytometry. (**A**) Representative density plots and histogram plots (red, IL-21; pink, IL-17A; black, isotype controls) of the staining are shown. (**B**) Expression (ΔMFI) levels of IL-21 and IL-17A are presented as mean ± SD of quadruplicate samples. Results shown are representative of two independent experiments. ****$p < 0.0001$. ns, not significant.

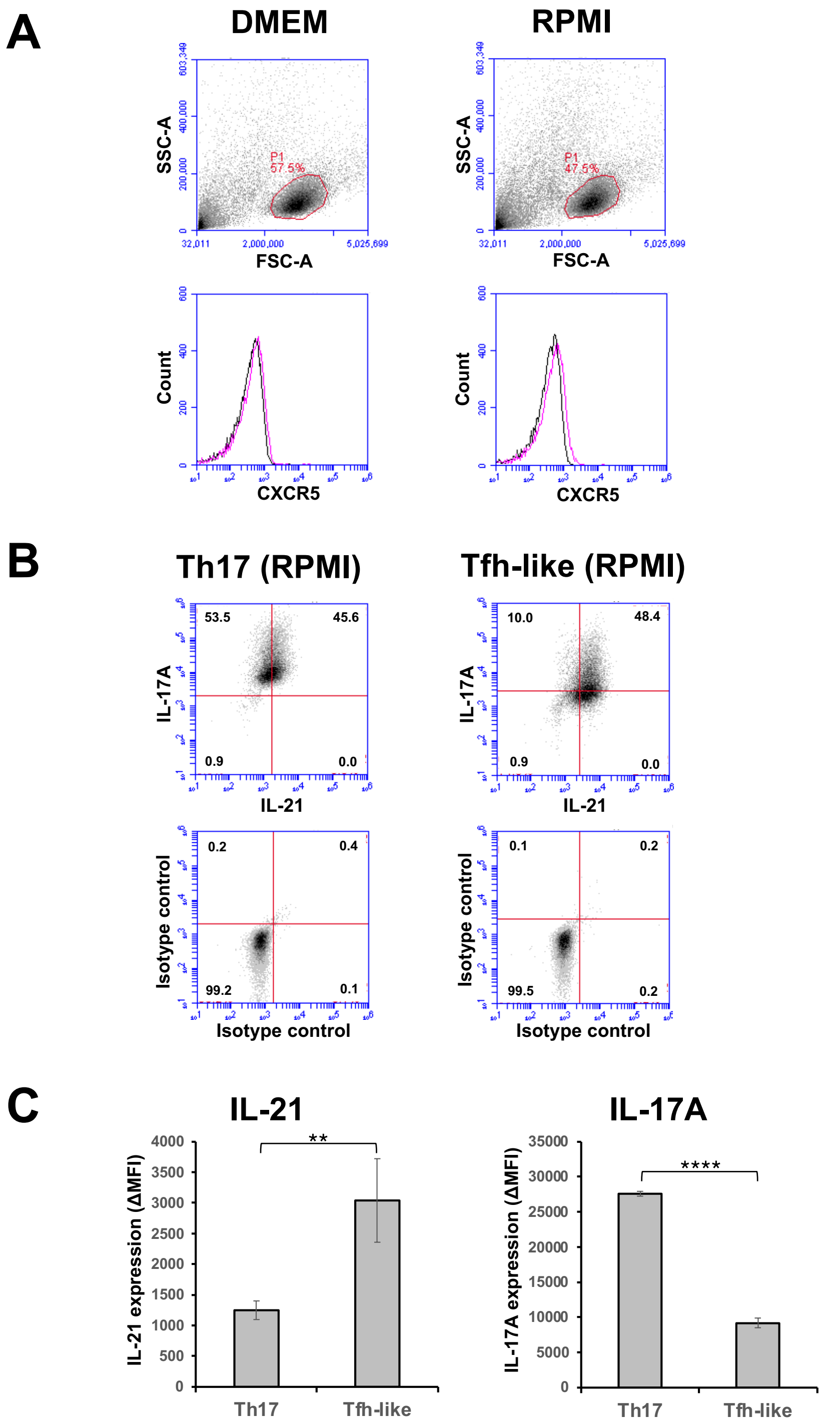

S7 Fig. The Tfh-like cells generated in the RPMI 1640 medium express slightly higher levels of CXCR5 expression than those generated in the DMEM, and produce higher levels of IL-21 and lower levels of IL-17A than Th17 cells. Tfh-like cells were induced as described in the legend of Fig 5 using either DMEM or RPMI 1640 medium. (**A**) Representative flow cytometry dot plots of FSC-A/SSC-A intensities and histogram plots of CXCR5 expression (pink, CXCR5; black, isotype controls) are shown. (**B**) Th17 cells and Tfh-like cells generated in the RPMI 1640 medium were restimulated with immobilized mAbs to CD3 and CD28 for 2 days, and monensin was added for the last 2 hours. The cells were fixed and permeabilized, and their intracellular IL-21 and IL-17A expression was analyzed by flow cytometry. Representative density plots of Th17 cells and Tfh-like cells are shown. (**C**) The expression levels of IL-21 and IL-17A are presented as mean ± SD of triplicate samples. Results shown are representative of two independent experiments. $^{**}p < 0.01$, $^{****}p < 0.0001$.

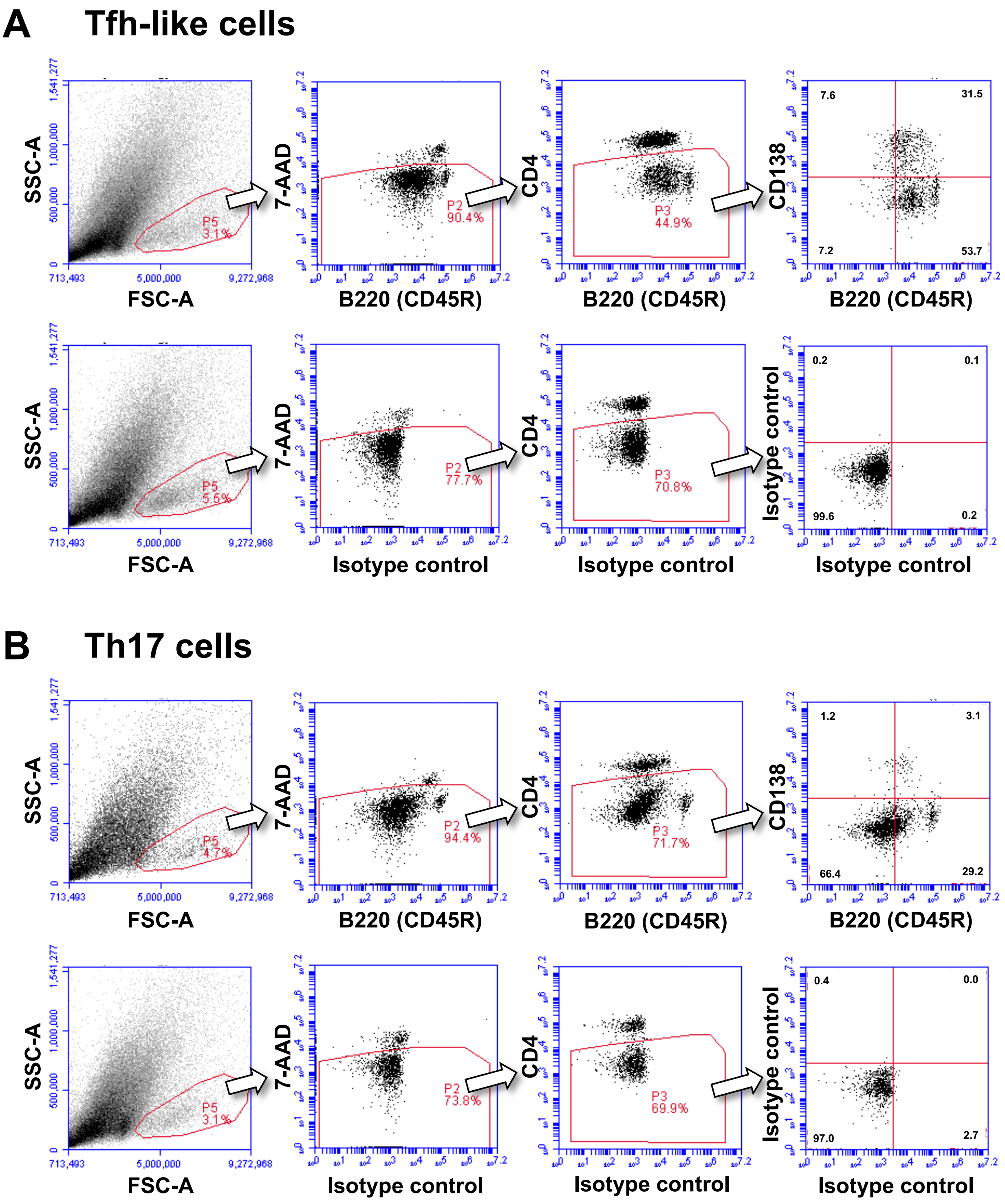

S8 Fig. The Tfh-like cells induce CD138^{high}B220^{low} plasma cells more efficiently than Th17 cells. Tfh-like cells and Th17 cells were induced from native CD4^{+} T cells in the RPMI medium as described in the Materials and Methods. These T cells were treated with mitomycin C and cultured with purified B cells (1:1 T/B ratio) for 7 days. The cultured cells were analyzed for their expression of the plasma cell markers CD138 and B220 by flow cytometry. Live cells were gated by using FSC-A/SSC-A dot plots and excluding 7-AAD^{+} cells, and CD4^{+} cells were excluded. The gating procedure of the cells are illustrated by using representative dot plots of the culture with Tfh-like cells (**A**) and Th17 cells (**B**).

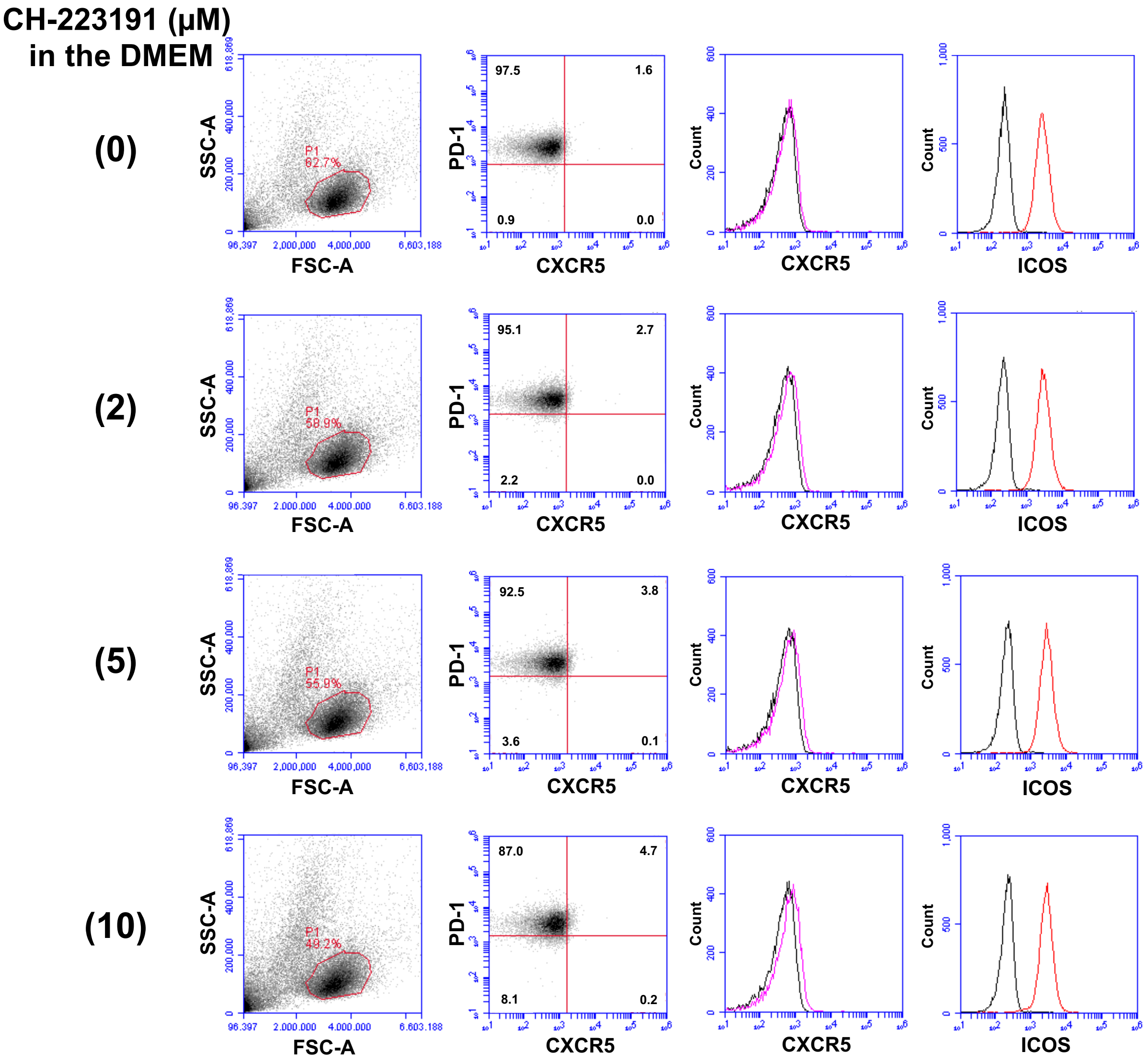

S9 Fig. Representative flow cytometry plots for Fig 6. Tfh-like cells were induced from naïve $CD4^+$ T cells in the presence of graded concentrations of CH-223191 in DMEM as described in the legend of Fig 6. Representative flow cytometry plots for their cell surface CXCR5, PD-1, and ICOS expression are shown (density plots for CXCR5/PD-1 expression and histogram plots for CXCR5 and ICOS expression). Results shown are representative of at least three independent experiments.

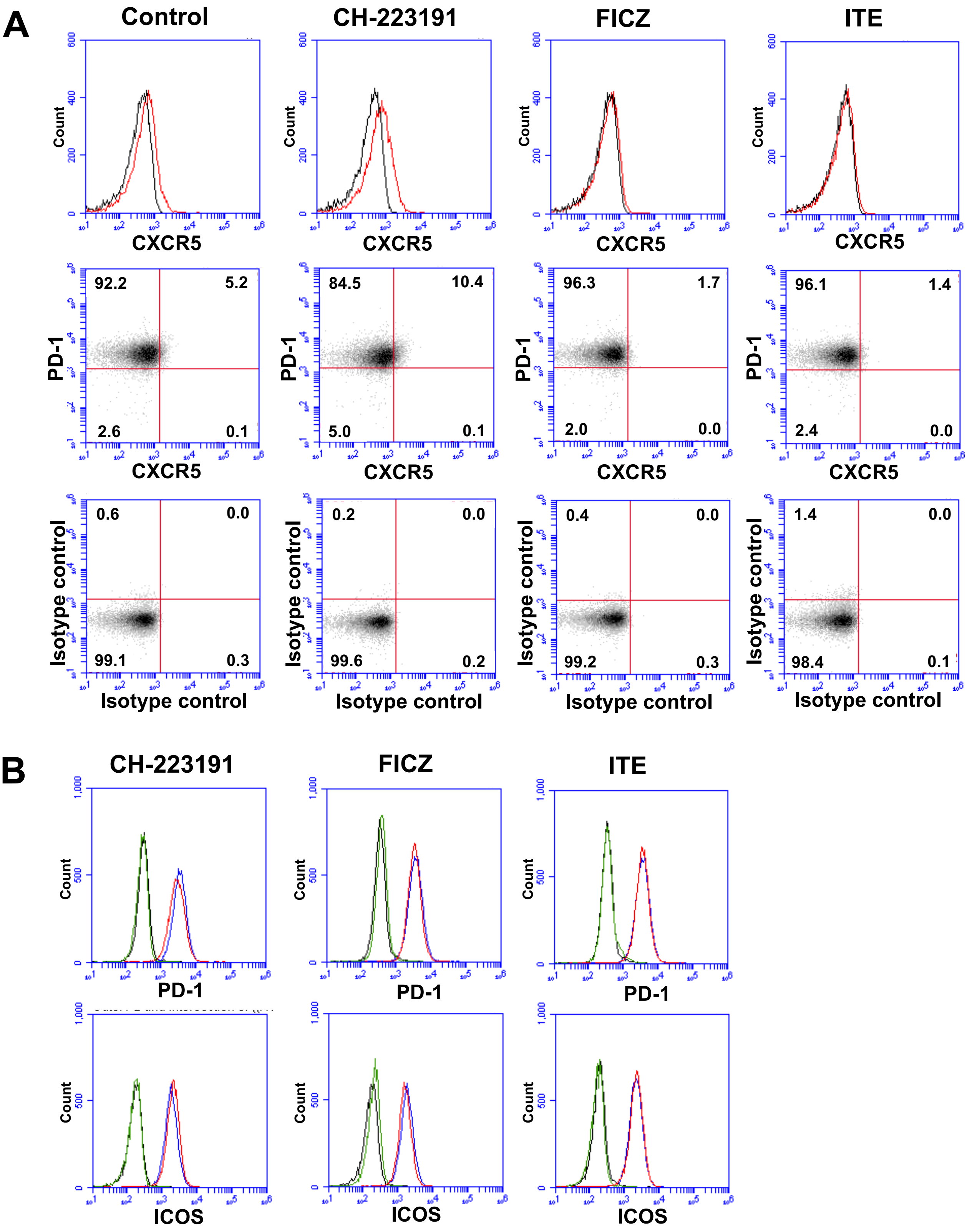

S10 Fig. Effects of AhR modulators on the expression of CXCR5, PD-1, and ICOS in Tfh-like cells. Naïve $CD4^{+}$ T cells were cultured under the "3+2d" condition in the RPMI 1640 medium. CH-223191 (5 μM), FICZ (100 nM), or ITE (1 μM) was added in the first culture. (**A**) Representative flow cytometry histograms of CXCR5 expression (red, APC-anti-CXCR5; black, APC-isotype control) and density plots for CXCR5 and PD-1 expression are shown. (**B**) Representative flow cytometry histograms of PD-1 and ICOS expression in the cells cultured with the indicated AhR modulator (red, FITC-anti-PD-1 or PE-anti-ICOS; green, isotype control) and those in the cells cultured without the modulator (blue, FITC-anti-PD-1 or PE-anti-ICOS; black, isotype control). Results shown are representative of at least three independent experiments.

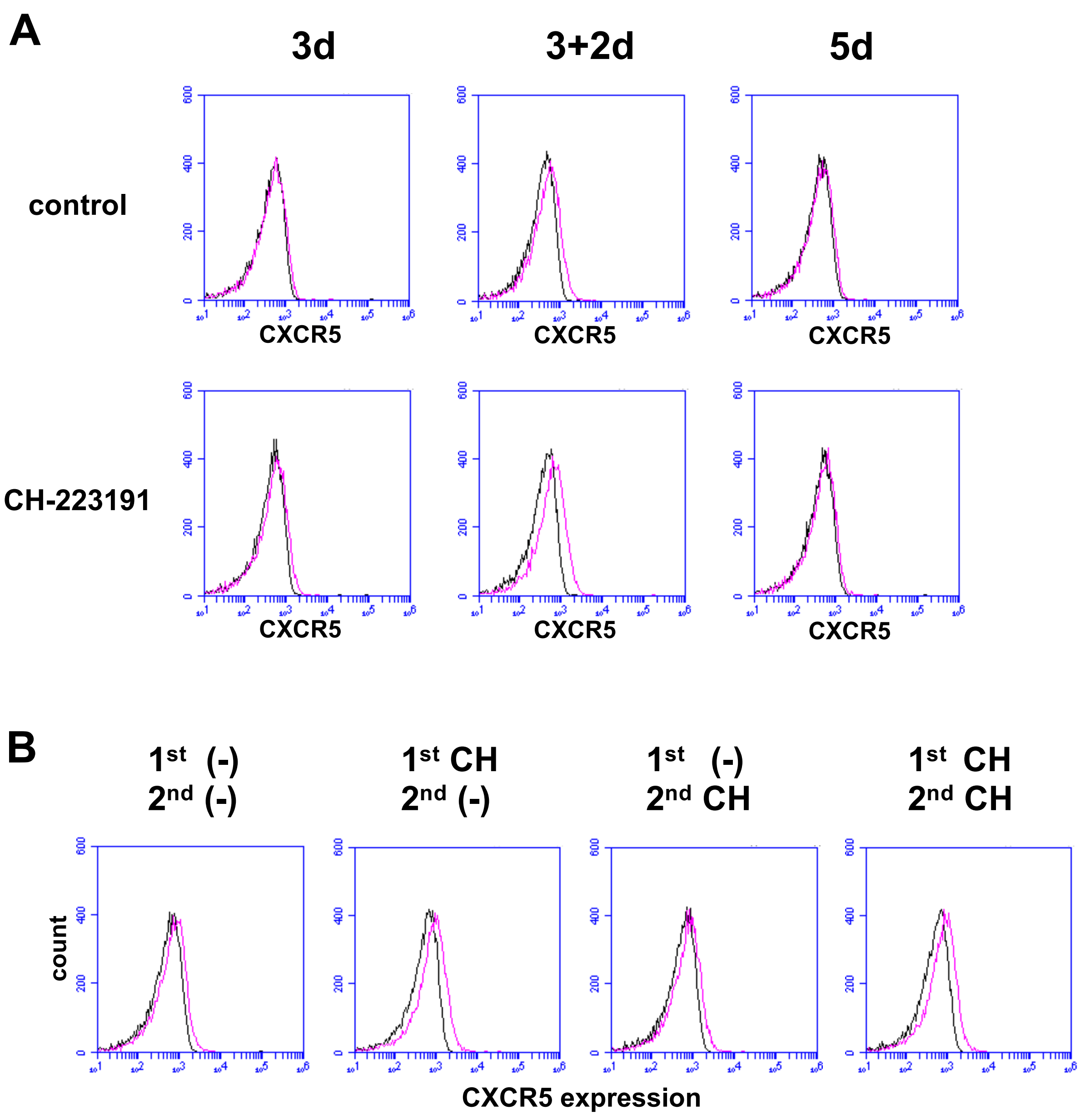

S11 Fig. The effect of CH-223191 on the expression of CXCR5 in Tfh-like cells. Naïve CD4$^+$ T cells were cultured under the "3+2d" Tfh-like cell condition in the RPMI 1640 medium. CH-223191 (5 μM) was added in the first culture and/or the second culture. (**A**) Representative flow cytometry histograms for the CXCR5 expression in Fig 8A (**A**) and Fig 8B (**B**) are shown (red, APC-anti-CXCR5; black, APC-isotype control). Results shown are representative of three independent experiments.

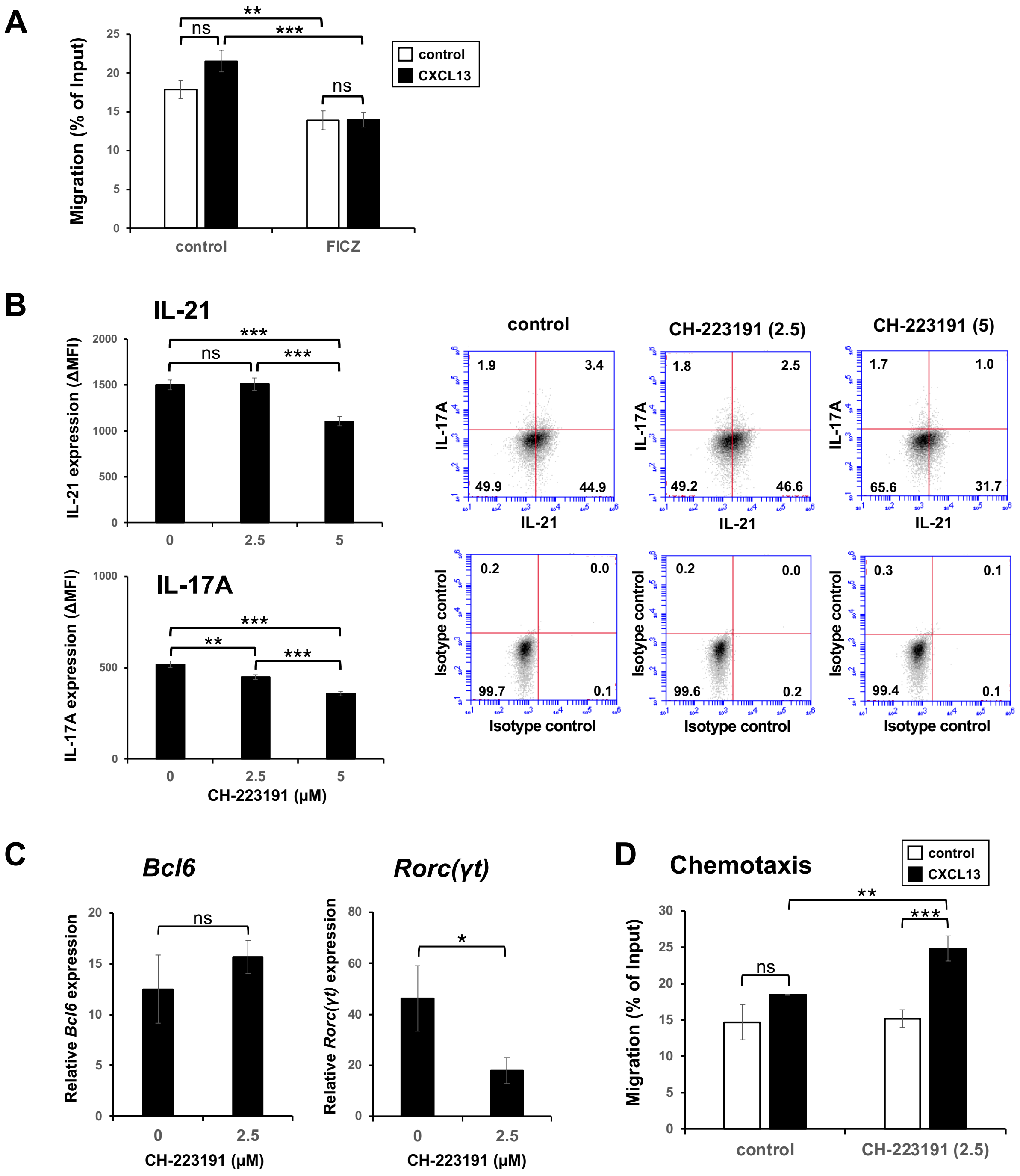

S12 Fig. FICZ-treated cells do not exhibit enhanced chemotaxis toward CXCL13, and CH-223191-treated Tfh-like cells produce IL-21. (**A**) Tfh-like cells were generated under the "3+2d" condition in the RPMI medium. FICZ (100 nM) or vehicle control was added in the first culture, and their chemotactic activity toward CXCL13 was assessed. (**B**, **C,** and **D**) Tfh-like cells were generated in the DMEM. CH-223191 (2.5 or 5 μM) or vehicle control was added in the first culture. After the "3+2d" culture, aliquots of the cells were restimulated with immobilized mAbs to CD3 and CD28 for 2 days in the RPMI medium, and monensin was added for the last 2 hours. Their intracellular IL-21 and IL-17A expression was analyzed by flow cytometry. The expression levels (ΔMFI) of IL-21 and IL-17A and representative density plots are shown (B). Immediately after the "3+2d" culture, aliquots of the cells were analyzed for *Bcl6* and *Rorc(γt)* expression by real-time PCR (C) or chemotaxis toward CXCL13 (D). Results are presented as mean ± SD of triplicate samples. *$p < 0.05$, **$p < 0.01$, ***$p < 0.001$. ns, not significant.

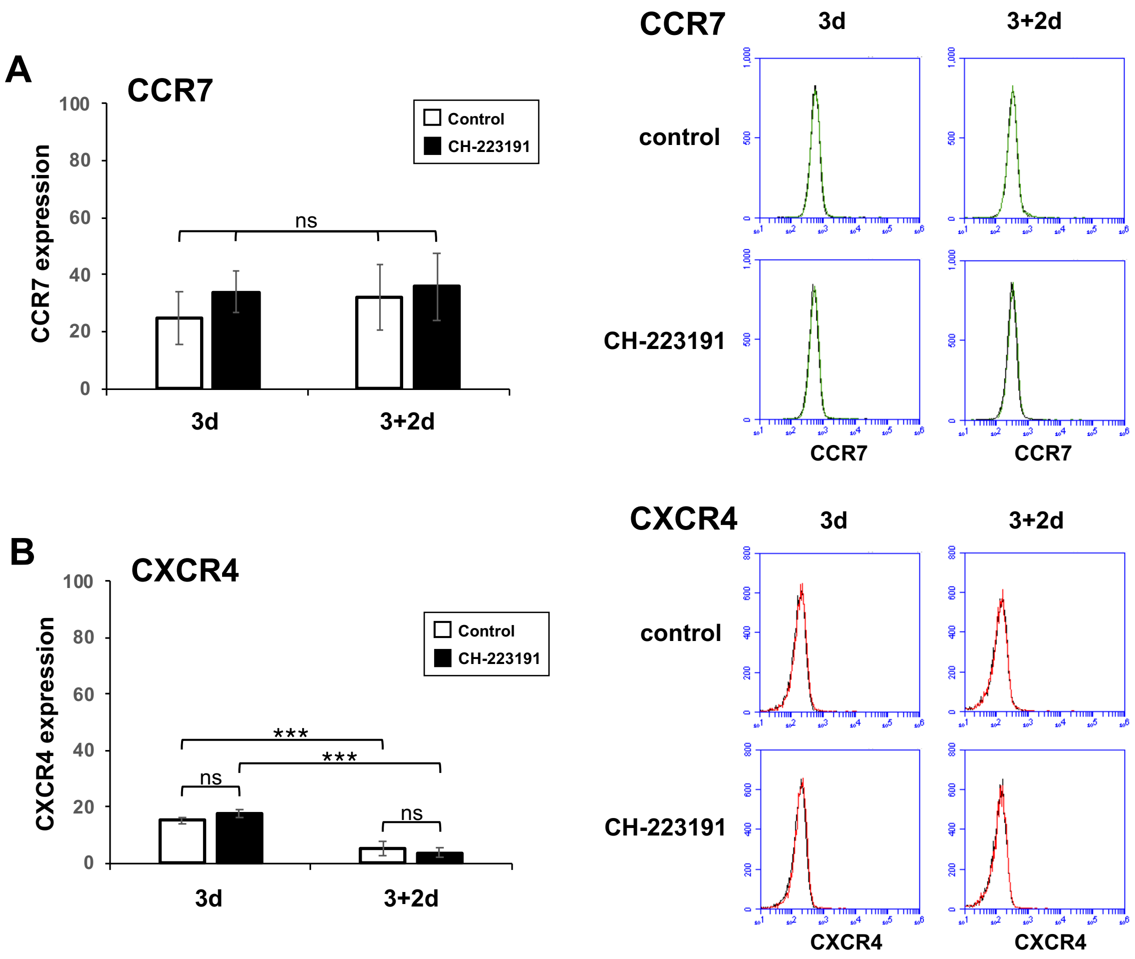

S13 Fig. Expression of CCR7 and CXCR4 chemokine receptors is low and unaffected by the addition of CH-223191. Naïve CD4$^+$ T cells were cultured as described in the legend of Fig 6 in the presence of CH-223191 (5 μM) or vehicle control. Representative flow cytometry histogram plots are shown. Data are presented as mean ± SD of samples (n = 3 for "3d", n = 4 for "3+2d"). ***$p < 0.001$, determined by one-way ANOVA with Tukey-Kramer multiple comparison test. ns, not significant.

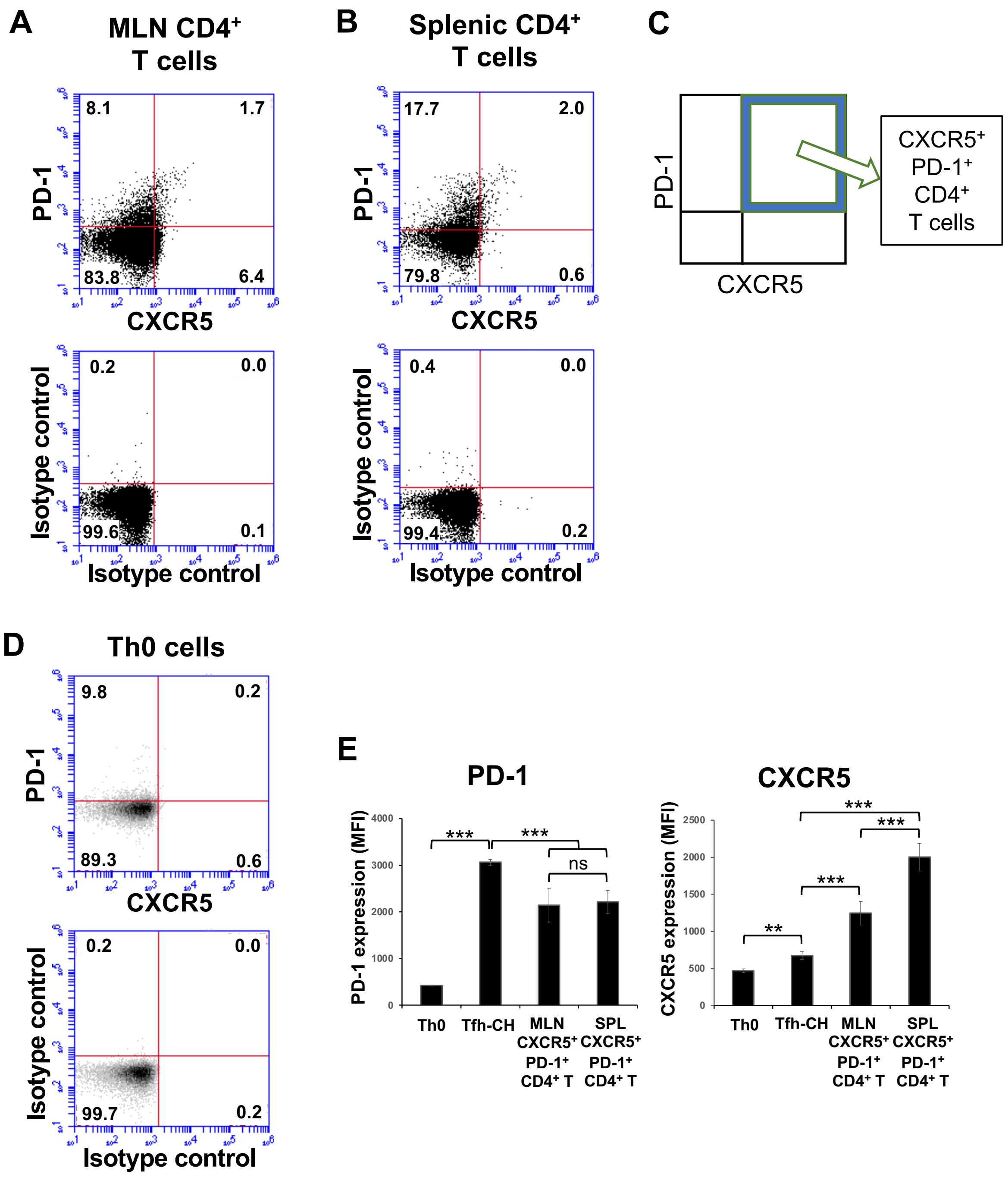

S14 Fig. Comparison of the expression levels of CXCR5 and PD-1 in Tfh cells from mesenteric lymph nodes (MLN) and spleens (SPL) of OVA-immunized mice and those in CH-223191-treated Tfh-like cells. Representative flow cytometry dot plots of CXCR5 and PD-1 expression in $CD4^+$ T cells from (**A**) MLN and (**B**) SPL are shown. (**C**) Gating strategies for Tfh cells ($CXCR5^+ PD\text{-}1^+ CD4^+$ cells) in $CD4^+$ T cells from MLN and SPL are illustrated. Representative flow cytometry density plots of CXCR5 and PD-1 expression in CH-223191-treated Tfh-like cells are shown in S10A Fig, and those in Th0 cells are shown in (**D**) as control. (**E**) Mean fluorescence intensities (MFI) of CXCR5 and PD-1 expression in MLN and splenic Tfh cells gated as in (C) and those in whole live Th0 and CH-223191-treated Tfh-like cells are shown. Data are presented as mean ± SD of three to seven samples. ***$p < 0.001$, **$p < 0.01$, ns, not significant. Results shown are representative of at least three independent experiments.
